# Signaling cascades in the failing heart and emerging therapeutic strategies

**DOI:** 10.1038/s41392-022-00972-6

**Published:** 2022-04-23

**Authors:** Xin He, Tailai Du, Tianxin Long, Xinxue Liao, Yugang Dong, Zhan-Peng Huang

**Affiliations:** 1 Department of Cardiology, Center for Translational Medicine, Institute of Precision Medicine, The First Affiliated Hospital, Sun Yat-sen University, Guangzhou, 510080 China; 2 NHC Key Laboratory of Assisted Circulation (Sun Yat-sen University), Guangzhou, China; 3 National-Guangdong Joint Engineering Laboratory for Diagnosis and Treatment of Vascular Diseases, Guangzhou, 510080 China

**Keywords:** Cardiology, Cardiovascular diseases

## Abstract

Chronic heart failure is the end stage of cardiac diseases. With a high prevalence and a high mortality rate worldwide, chronic heart failure is one of the heaviest health-related burdens. In addition to the standard neurohormonal blockade therapy, several medications have been developed for chronic heart failure treatment, but the population-wide improvement in chronic heart failure prognosis over time has been modest, and novel therapies are still needed. Mechanistic discovery and technical innovation are powerful driving forces for therapeutic development. On the one hand, the past decades have witnessed great progress in understanding the mechanism of chronic heart failure. It is now known that chronic heart failure is not only a matter involving cardiomyocytes. Instead, chronic heart failure involves numerous signaling pathways in noncardiomyocytes, including fibroblasts, immune cells, vascular cells, and lymphatic endothelial cells, and crosstalk among these cells. The complex regulatory network includes protein–protein, protein–RNA, and RNA–RNA interactions. These achievements in mechanistic studies provide novel insights for future therapeutic targets. On the other hand, with the development of modern biological techniques, targeting a protein pharmacologically is no longer the sole option for treating chronic heart failure. Gene therapy can directly manipulate the expression level of genes; gene editing techniques provide hope for curing hereditary cardiomyopathy; cell therapy aims to replace dysfunctional cardiomyocytes; and xenotransplantation may solve the problem of donor heart shortages. In this paper, we reviewed these two aspects in the field of failing heart signaling cascades and emerging therapeutic strategies based on modern biological techniques.

## Introduction

Cardiovascular disease has become one of the heaviest health burdens worldwide. Approximately 40% of the total deaths worldwide are attributed to cardiovascular disease, which is the “No. 1 killer” that claims more than 17 million lives each year.^1^ Due to the great effort that has been put into improving the prognosis of cardiovascular diseases, the incidence of chronic heart failure, which is the end stage of most cardiovascular diseases, has gradually decreased over time. However, in a population-based study of 4 million individuals in the UK, approximately 3 out of 1000 patients developed heart failure each year.^2^ The prognosis associated with chronic heart failure is still relatively poor. At 1, 5, 10, and 15 years after the diagnosis of chronic heart failure, the survival rates were 75.9%, 45.5%, 24.5%, and 12.7%, respectively,^3^ which significantly affected the life expectancy of these patients. To make matters worse, chronic heart failure is also characterized by a high risk of recurrent and worsening episodes. Recurrent emergency department visits and hospitalizations significantly add to the economic burden. In 2014, in the United States, it was estimated that a total of 11 billion dollars were spent on heart failure hospitalizations.^4^ In Denmark, a chronic heart failure patient has to spend an average of 17,039 euros per year for this disease, which is nearly 3 times the cost of a control individual matched by age, gender, marital status, and municipality.^5^ Therefore, improving the survival of chronic heart failure patients is of paramount importance in global public health and the economy.

With the development of genetic techniques, we have a much deeper understanding of the signaling cascades associated with the chronic heart failure. However, translational work, to some extent, is still lagging behind. The standard chronic heart failure treatments, which are angiotensin II-converting-enzyme inhibitors/angiotensin II receptor blockers, beta-blockers, and aldosterone receptor antagonists, were established more than 10 years ago.^6–12^ The pathophysiology underlying these therapies, neurohormonal activation in chronic heart failure, was discovered even longer ago. Recent breakthroughs in chronic heart failure treatment are also based on outdated mechanistic discoveries. Ivabradine slows the heart rate by blocking the funny channel in the sinoatrial node,^13^ which was discovered in 1979.^14^ Sacubitril–valsartan provided additive survival benefits for chronic heart failure patients by further inhibiting the neutral endopeptidase neprilysin.^15^ The great success of sodium–glucose cotransporter 2 inhibitors in chronic heart failure^16,17^ originated from an unexpected observation of heart failure risk reduction in clinical trials for diabetes.^18^ The molecular mechanism underlying the benefit of sodium–glucose cotransporter 2 inhibitor treatment remains unclear.

Therapeutic improvement is always derived from novel achievements in mechanistic or technical research. Therefore, to provide insights for future translational efforts, we believe it is important to review these achievements. In this paper, we reviewed signaling cascades involved in failing hearts and emerging therapies based on modern biological techniques.

## Signaling cascades in failing heart

**Fig. 1:**
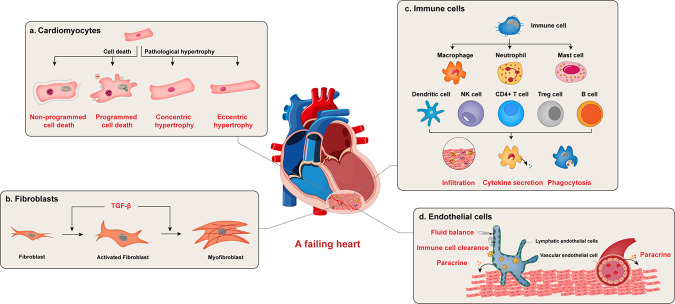
Functions of different cell types in a failing heart. Heart failure is a complex process that involves multiple cell types in the heart. Under stress, cardiomyocytes undergo either pathological hypertrophy or cell death. Hypertrophy led to cardiomyocyte dysfunction, while non-programmed or programmed cell death led to cardiomyocyte loss. Cardiac fibrosis is another form of cardiac remodeling. It mainly involves fibroblast activation and conversion to myofibroblast. Various immune cells also contribute to heart failure. These cells infiltrate the injured myocardium, secret cytokines, and cleared unwanted material to regulate inflammation, regeneration, and function of other cell types in the failing heart. Both vascular endothelial cells (VECs) and lymphatic endothelial cells (LECs) regulate cardiac function. VECs affect neighboring cardiac cells by paracrine factors. LECs regulate cardiac regeneration after infarction by maintaining fluid balance, promoting immune cell clearance, and also secreting paracrine factors

With achievements in molecular studies, we will have many more therapeutic targets and opportunities for heart failure treatments. As ischemic cardiomyopathy and myocardial infarction are the most prevalent causes of chronic heart failure, the mechanisms of these diseases and myocardial regeneration are closely related to heart failure. Recent progress has shown that in addition to cardiomyocytes and fibroblasts, immune cells, microvascular endothelial cells, and lymphatic endothelial cells are important players in maintaining normal cardiac function and chronic heart failure pathophysiology (Fig. 1). The signal transduction in these cells in failing heart will be discussed individually. Heart failure with preserved ejection fraction (HFpEF) has gained increasing attention in recent years. Although in the same entity of heart failure, there are substantial differences in epidemiology, pathophysiology, and most importantly, responses to heart failure medication between HFpEF and heart failure with reduced ejection fraction (HFrEF). Therefore, studies focusing on the molecular mechanism of this chronic heart failure subtype will be briefly reviewed.

### Cardiomyocytes

#### Pathological cardiomyocyte hypertrophy

Under mechanical or biochemical stress, the heart undergoes adaptive remodeling to maintain cardiac output and systemic perfusion. Pathological cardiomyocyte hypertrophy is one of these remodeling processes and is characterized by an increase in cardiomyocyte mass, sarcomere rearrangement, and fetal gene reactivation. Although pathological cardiomyocyte hypertrophy is considered a compensatory mechanism at the beginning of stress, prolonged and uncontrolled hypertrophy leads to chronic heart failure.^19^ A better understanding of the mechanism of pathological cardiomyocyte hypertrophy would help identify potential therapeutic targets for chronic heart failure. Except for the canonical protein-based signaling cascades, noncoding RNAs and RNA modifications are also extensively involved in the development of pathological cardiomyocyte hypertrophy.

##### Canonical signaling pathways

**Calcineurin-nuclear factor of activated T cells (NFAT) signaling**: Calcineurin is a calcium- and calmodulin-dependent serine/threonine protein phosphatase that is a heterodimer composed of the catalytic subunit calcineurin A (CnA) and the regulatory subunit calcineurin B (CnB). The C-terminus of CnA is an autoinhibitory domain that forms α-helices to block the upstream catalytic site under basal conditions. When there is an increase in the intracellular Ca^2+^ concentration, Ca^2+^ occupies low-affinity binding sites in CnB, which in turn leads to a conformational change in CnA. This change promotes the binding of Ca^2+^-calmodulin. The interaction between Ca^2+^ and CnA releases the catalytic site from the autoinhibitory domain, and the calcineurin complex becomes fully activated.^20^ This process couples calcium signaling to the dephosphorylation of multiple downstream substrates of calcineurin, one of which is NFAT. The subcellular localization of NFAT largely depends on its phosphorylation status. Once dephosphorylated by calcineurin, NFAT translocates from the cytoplasm to the nucleus and acts as a transcription factor to initiate the transcription of multiple genes,^20^ including those that promote pathological cardiomyocyte hypertrophy. In 1988, Molkentin et al. generated a transgenic mouse that overexpressed a constitutively active form of CnA lacking the C-terminal autoinhibitory domain in cardiomyocytes.^21^ Every transgenic mouse spontaneously developed severe cardiac hypertrophy, which could be prevented by the calcineurin inhibitor cyclosporin A. Calcineurin inhibition also attenuated phenylephrine (PE)- or angiotensin II (Ang II)-induced pathological cardiomyocyte hypertrophy in vitro. As NFAT3 is the dominant NFAT isoform in the heart,^22^ a transgenic mouse with constitutively active NFAT3 in cardiomyocytes was also generated. NFAT3 transgenic mice successfully recapitulated the hypertrophy phenotype of CnA transgenic mice. Since then, much effort has been put into treating cardiac hypertrophy by calcinurin inhibition. Transgenic mice overexpressing dominant-negative mutants of calcineurin or inhibitory proteins downregulated calcineurin signaling. These mice were protected from pressure overload- or isoproterenol infusion-induced cardiac hypertrophy^23–25^. Genetic deletion of the calcineurin partner Ca^2+^ and integrin-binding protein-1 (CIB1) also reduced activity of calcineurin. CIB1-KO mice developed less severe pathological hypertrophy induced by pressure overload than control mice. In contrast, physiologic hypertrophy induced by swimming activity was not altered.^26^ Despite these promising results, some studies raised concerns about calcineurin inhibition. Overexpressing the endogenous inhibitor of calcineurin ZAKI-4 beta attenuated pressure overload-induced cardiac hypertrophy but also exacerbated diastolic dysfunction.^27^ Another study showed that different CnA isoforms had different effects. CnA is encoded by 3 genes (CnAα, CnAβ, and CnAγ). CnAβ is dominant in the heart. Two spliced variants of CnAβ have been identified (CnAβ1 and CnAβ2). Specifically, CnAβ2 differs from typical CnA in that the C-terminal autoinhibitory domain is replaced by a unique region with an unknown function.^28^ Interestingly, CnAβ2 overexpression protected the heart from pressure overload-induced hypertrophy and myocardial infarction. This protective effect was not dependent on NFAT dephosphorylation. Instead, Akt/mammalian target of rapamycin (mTOR) might be involved.^23,29^

**G protein-coupled receptor-mediated signaling**: It is well established that endothelin-1 (ET-1), Ang II, and catecholamines are important inducers of cardiac hypertrophy and chronic heart failure. These extracellular signals are transduced to intracellular effectors mostly through G protein-coupled receptors.^30^ These receptors are transmembrane proteins that possess a G protein binding site on the intracellular domain. Once bound to a ligand, a G protein-coupled receptor undergoes a conformational change and acts as a guanine nucleotide exchange factor to replace GDP with GTP on a G protein. This process activates the G protein by releasing the α subunit (Gα) from the β and γ subunits (Gβγ).^31^ The activated protein G stimulates various downstream signaling pathways, depending on the type of Gα. ET-1, angiotensin II receptors, and α-adrenergic receptors are coupled to Gq/11. Activated Gq/11 binds to phospholipase Cβ (PLCβ), which induces the generation of diacyl glycerol (DAG) and inositol-1,4,5-trisphosphate (IP3). The former activates protein kinase C (PKC), and the latter triggers an increase in intracellular Ca^2+^. Increased Ca^2+^ can activate not only calcineurin-NFAT signaling but also calmodulin-dependent kinase (CamK). CamK phosphorylates histone deacetylases (HDACs) and promotes shuttling from the nucleus to the cytoplasm.^32^ HDAC represses transcriptional activity through histone deacetylation. Under basal conditions, HDAC binds to the transcription factor myocyte enhancer factor-2 (MEF2) and suppresses its activity. MEF2 is released when HDAC leaves the nucleus and activates the transcription of hypertrophic genes.^33^ Gαq overexpression is sufficient to induce cardiac hypertrophy.^34–36^ Genetic deletion of Gαq/Gα11 or overexpression of its inhibitory peptide blocks cardiac hypertrophy induced by pressure overload.^37–39^

β1-adrenergic receptor is coupled to Gs, which activates adenylate cyclase and downstream cAMP/protein kinase A (PKA) signaling.^40–42^ β1-adrenergic receptor is believed to mediate the positive chronotropic, inotropic, and lusitropic effects of catecholamines. In vivo overexpression of β1-adrenergic receptor, Gs, or downstream PKA leads to cardiac hypertrophy and ultimately heart failure.^43–46^

Regulators of G protein signaling (RGSs) are a family of proteins that share a homologous RGS or RGS-like domain. The subfamilies R4/B, R7/C, R12/D, and RZ/A are highly expressed in the heart. Most RGSs inhibit G proteins by promoting the intrinsic GTPase activity of Gα,^47^ although various other functions have also been documented. RGS2 deficiency leads to a more severe cardiac hypertrophic phenotype induced by pressure overload, and decreased Gαq signaling, but it did not affect exercise-induced cardiac hypertrophy because Gαq activation was not involved.^48^ RGS14 also inhibited hypertrophy. Interestingly, this effect appeared to be mediated by crosstalk with mitogen-activated protein kinase (MEK)-extracellular signal-regulated protein kinase (ERK) 1/2 signaling. In contrast, a study showed that RGS12 promoted cardiac hypertrophy by activating MEK-ERK1/2 signaling.^49^

**Mitogen-activated protein kinase (MAPK) signaling**: In mammalian cells, 3 families of MAPKs have been identified: extracellular responsive kinases (ERKs), c-Jun N-terminal kinases (JNKs), and p38 MAPKs.^50^ Each of the MAPK families is part of a protein kinase cascade consisting of a sequence of kinases that are activated in series: a MAPK kinase kinase (MAPKKK), a MAPK kinase (MAPKK) and a MAPK. MAPKKK can directly sense stretch or can be activated by an upstream MAPKKK kinase (MAPKKKK) or a small G protein. The MAPK pathway can transduce multiple extracellular signals through various receptors, such as hypertrophic signals mediated by G protein-coupled receptors, transforming growth factor-β signals mediated by receptor serine/threonine kinases, and insulin-like growth factor-I (IGF-1) signals mediated by receptor tyrosine kinase.^51^ Once activated, MAPKs phosphorylate their downstream transcription factors to regulate their activity.

**ERK signaling**: There are 5 subtypes of ERK, among which ERK1 and ERK2 are the most widely studied. Corresponding MAPKKs upstream of ERK1/2 are MEK1/2. Upstream of MEK1/2 is a MAPKKK called RAF1 that is activated by a small G protein in the Ras family. Once activated, ERK1/2 translocates to the nucleus and phosphorylates multiple transcription factors, such as cAMP-responsive element-binding protein (CREB) and ELK1. Activation of the ERK1/2 MAPK cascade ultimately leads to a transcriptomic change that favors cell growth.^50^ Transgenic mice with cardiomyocyte-specific activated MEK1 overexpression developed spontaneous cardiac hypertrophy. MEK1 specifically activated ERK1/2 but not p38 or JNK. Interestingly, cardiac function was increased in these mice without signs of decompensation over time. Cardiomyocytes with MEK1 overexpression were resistant to apoptotic stimuli both in vitro and in vivo. These data indicated that the ERK1/2 pathway was associated with concentric physiologic but not pathologic hypertrophy.^52^ In addition to classic RAF1-MEK1/2 signaling, ERK1/2 can also incorporate signaling from other pathways. MEK1/2 activates ERK1/2 by phosphorylating threonine and tyrosine in the threonine-glutamate-tyrosine (TEY) motif within the activation loop, which are Thr183 and Tyr185 in murine ERK2.^53^ A study showed that ERK2 could also be autophosphorylated at Thr188, which requires an interaction between ERK2 and Gβγq. A gain-of-function mutation in ERK2 that mimics phosphorylation promoted pressure overload-induced cardiac hypertrophy, while a loss-of-function mutation attenuated this phenotype. A mechanistic study showed that the ERK2 Thr188 mutation did not alter the phosphorylation of cytosolic substrates of ERK1/2 but dramatically changed the phosphorylation of its nuclear targets, suggesting that autophosphorylation at Thr188 promotes ERK2 nuclear translocation.^54^ In contrast, cardiomyocyte-specific activation of MEK5-ERK5 led to lethal dilated cardiomyopathy. Although the fetal gene program was activated, the relative heart weight was not increased. Cardiomyocytes in these mice had an elongated morphology with a decreased cross-sectional area. These data suggested that ERK5 was responsible for pathological eccentric cardiac hypertrophy.^55^

**p38 signaling**: p38 signaling can be induced by multiple stress and inflammatory stimuli, such as oxidative stress, infection, and cytokines. MEK3 and MEK6 are the two important activators of p38, which in turn can be activated by a MAPKKK called TGF-beta activated kinase (TAK). Activated p38 phosphorylates several hypertrophic transcription factors, such as MEF2.^56^ TAK1 and p38 activation can be observed in cardiac hypertrophy models induced by pressure overload, ET-1 or PE.^57–60^ Overexpression of an activated TAK1 mutant in cardiomyocytes led to p38 phosphorylation in vivo, cardiac hypertrophy, fibrosis, and eventually heart failure.^60^ Dual-specificity protein phosphatases (DUSPs) are a family of specialized phosphatases that can dephosphorylate MAPKs and inactivate them.^61^ Among them, DUSP1 and DUSP4 mainly act on p38. DUSP1 and DUSP4 double-deficient mice developed severe dilated cardiomyopathy and cardiac hypertrophy, which could be rescued by pharmacological inhibition of p38.^62^ The function of MEK3/6 in cardiac hypertrophy remains controversial. In vitro overexpression of MEK3/6 in cardiomyocytes induced hypertrophic responses, including cell enlargement, sarcomere reorganization, and increased ANP expression.^63^ However, an in vivo study told a different story. In vivo overexpression of MEK3 and MEK6 resulted in p38 activation, interstitial fibrosis and fetal gene expression. The transgenic heart had both systolic dysfunction and restrictive diastolic abnormalities. However, heart weight was not significantly changed. Examination of cross-sections of the heart suggested that MEK3 overexpression led to heterogeneous myocyte atrophy and sporadic hypertrophy, while MEK6 overexpression only resulted in moderate cellular hypertrophy.^64^ Inconsistent results were reported by another study. Mice expressing dominant-negative MEK3 and MEK6 developed cardiac hypertrophy at baseline and had more severe cardiac hypertrophy induced by pressure overload, Ang II, isoproterenol or PE than control mice. Augmentation of calcineurin-NFAT signaling might mediate the p38 inhibition-associated phenotype.^65^ These inconsistent data indicate that the association between p38 signaling and hypertrophic heart growth is complex. There are 4 p38 MAPKs: p38α, p38β, p38γ, and p38δ, which can be grouped into two subsets based on structural similarity.^66^ Each isoform appears to have a distinct role in cardiac hypertrophy. Inhibiting p38α by overexpressing a dominant-negative mutant induced spontaneous cardiac hypertrophy and promoted hypertrophic changes in response to stimulation.^65^ The right ventricle (RV) and left ventricle (LV) undergo distinct postnatal growth that results in a larger LV relative to the RV. Cardiomyocytes in the RV had lower proliferation, more apoptosis, and a smaller average size than cardiomyocytes in the LV, which was accompanied by selective activation of p38 in the RV. Genetic deletion of p38α and p38β led to enlargement in the RV but not the LV, suggesting that selective p38 activation was important for proper chamber organization during development.^67^ In contrast to p38α-deficient mice, p38γ- and p38δ-deficient mice had impaired postnatal hypertrophic heart growth, which eventually led to a smaller heart. Under Ang II stimulationA, there was also no obvious hypertrophic heart growth in these genetically deficient mice. A mechanistic study showed that p38γ and p38δ could phosphorylate the mTORC inhibitor DEPTOR and promote its degradation.^68^ Taken together, these data indicate that p38α and p38β inhibit cardiac hypertrophy, while p38γ and p38δ promote hypertrophy. The regulation of p38 MAPK signaling appears to involve a more complex network than a simple MAPKKK-MAPKK-MAPK cascade.

**JNK signaling**: Three JNK MAPKs have been identified: JNK1, JNK2, and JNK3. JNK1 and JNK2 are expressed in many cell types, while JNK3 is mainly expressed in the heart, nervous system and testis.^69^ Upstream MAPKKs mainly include MEK4 and MEK7. MAPKKKs that regulate MEK4/7 include MEKK1, MEKK2, MEKK3, and mixed lineage kinases 2 and 3 (MLK2 and MLK3). Activated JNK translocates into the nucleus and phosphorylates multiple transcription factors, such as c-JUN, activating transcription factor 2 (ATF-2), ELK-1, and p53. Overexpressing active MEK7 in neonatal rat cardiomyocytes induced hypertrophy.^70^ However, an in vivo study showed that MEK7 overexpression in adult mice led to severe heart failure, which was due to the downregulation of Cx43 and the loss of gap junctions, and no obvious cardiac hypertrophy was observed.^71^ A loss-of-function study showed that delivery of a dominant-negative MEK4 expression vector to the heart blocked pressure overload-induced JNK activation and cardiac hypertrophy.^72^ Genetic deletion of MEKK1 attenuated cardiac hypertrophy induced by Gαq overexpression. This deletion specifically blocked JNK activation but did not affect the phosphorylation of ERK or p38.^73^ However, another study showed that MEKK1 deficiency did not affect cardiac hypertrophy and exacerbated heart failure induced by pressure overload. This outcome was possibly due to increased levels of apoptosis and inflammation.^74^ Similarly, deletion of JNK1, JNK2, or JNK3 did not affect pressure overload-induced cardiac hypertrophy, but JNK1 deletion led to the rapid deterioration of cardiac function, which was associated with increased apoptosis and inflammatory infiltration in the heart.^75^ Another study indicated that JNK was a negative regulator of cardiac hypertrophy. Mice expressing dominant-negative JNK1 and JNK2 displayed increased cardiac hypertrophy induced by pressure overload. This effect could be mediated by crosstalk with the calcineurin/NFAT signaling pathway.^76^ Therefore, although JNK activation can be induced by cardiac stress, it is still unclear how JNK regulates cardiac hypertrophy.

**Phosphoinositide 3-kinases (PI3K)-AKT signaling**: PI3K is an important molecule that mediates signals of cell growth and proliferation. There are 4 classes of PI3Ks, among which Class I is further divided into the IA and IB subsets. PI3Ks can be regulated by tyrosine kinase receptors such as IGF-1 receptor and GRCPs, including α- and β-adrenergic receptors.^77–79^ Class IA PI3K is composed of a p110 catalytic subunit (with α, β, or δ isoforms) and a p85 regulatory subunit, while Class IB PI3K is composed of a catalytic p110γ and a regulatory p101 subunit. Tyrosine kinase receptors regulate Class IA PI3K, and GRCPs regulate Class IB PI3K. Once activated, PI3K catalyzes the conversion of phosphatidylinositol-4,5-bisphosphate (PIP2) to phosphatidylinositol-3,4,5-trisphosphate (PIP3). PIP3 directly binds protein kinase B (PKB)/AKT and phosphoinositide-dependent kinase-1 (PDK1). Because PIP3 is membrane-restricted, binding recruits both AKT and PDK1 to the membrane and promotes their interaction. The interaction of PDK1 and AKT leads to AKT phosphorylation and activation, and phosphorylated AKT in turn phosphorylates downstream effectors such as mTOR and glycogen synthase kinase 3β (GSK3β). Phosphorylated mTOR enhances protein synthesis in 2 ways: (1) by activating S6 kinase-1 and S6 kinase-2 and increasing ribosomal biogenesis and (2) by releasing eukaryotic initiation factor 4E (eIF4E) from its binding protein and promoting translation initiation.^80,81^ GSK3β negatively regulates hypertrophic transcription factors, such as c-JUN,^82^ c-MYC,^83^ STAT,^84^ NF-κB,^85^ NFAT,^86^ and GATA4.^87^ The phosphorylation of GSK3β attenuates its inhibitory activity.

Genetic deletion of the p85α isoform of the regulatory subunit of Class IA PI3K led to attenuated Akt signaling and reduced heart size. Exercise-induced cardiac hypertrophy was also attenuated in these mice.^88^ Overexpression of constitutively active p110α resulted in larger hearts, while the expression of dominant-negative p110α resulted in smaller hearts. Cardiomyocytes sizes changed accordingly. However, these changes in heart growth were not associated with cardiomyopathic phenotypes, such as myocyte necrosis, apoptosis, interstitial fibrosis or contractile dysfunction.^89^ Interestingly, IA PI3Kα appeared to protect the heart against pathological growth. Overexpression of active p110α improved the survival of a mouse model of dilated cardiomyopathy and reduced relative cardiac hypertrophy induced by pressure overload, while inhibiting p110α had the opposite effect. This effect might be mediated by the PI3Kα’s negative regulation of GPCR-induced activation of ERK1/2 and Akt.^90^ Phosphatase and tensin homolog (PTEN) counteracts PI3K activity. Inactivation of PTEN led to cardiac hypertrophy and a decrease in cardiac contractility. While the change in cardiomyocyte size was mediated by PI3Kα, decreased contractility was mediated by the activation of PI3Kγ. Deletion of p110γ enhanced cardiac contractility without obvious hypertrophy. The negative inotropic effect of PI3Kγ was mediated by inhibiting cAMP production.^91^ Another study provided further insights into the function of PI3Kγ. Although PI3Kγ-deficient mice had enhanced contractility at baseline, they developed severe heart failure after pressure overload. PI3Kγ^−/−^ mice had heart chamber dilation and an impaired compensatory hypertrophic response that was characterized by the absence of left ventricular wall thickening. Massive cardiac necrosis and secondary fibrosis were observed in the failing heart. However, inhibiting PI3Kγ kinase activity protected the heart from pathological hypertrophy induced by pressure overload without increasing myocardial damage. Mechanistic studies indicated that PI3Kγ functions via 2 distinct pathways. One pathway is kinase-dependent regulation of Akt and MAPK, which mediates cardiac hypertrophy. The other pathway is kinase-independent regulation of cAMP degradation by interacting with and controlling phosphodiesterase (PDE) 3b. The second pathway is believed to negatively regulate cardiac contractility.^92^

Overexpression of an active form of downstream Akt induced concentric cardiac hypertrophy, increased contractility and decreased diastolic function under stress. GSK3β phosphorylation was increased, which in turn mediated GATA4 translocation into the nucleus.^87^ Akt1-deficient mice were resistant to exercise- or IGF-1-induced cardiac hypertrophy but were sensitized to pressure overload- or ET-1-induced hypertrophy.^93^ These data indicated that Akt1 had differential regulatory effects on pathological and physiological cardiac hypertrophy. Interestingly, a study from Wang et al. indicated an opposite function of Akt1 in the nucleus. 11,12-regioisomeric epoxyeicosatrienoic acids (11,12-ETT) was an anti-hypertrophic metabolite. This effect was mediated by the its induction of accumulation of phospho-Akt1 in the nucleus. 11,12-ETT induced phosphor-Akt1 nucleus translocation by promoting interaction of AMP-activated protein kinase catalytic subunit alpha 2 (AMPKα2) with Akt1.^94^

Hypertrophic stimuli such as isoproterenol and PE led to GSK3β phosphorylation.^95^ Overexpression of active GSK3β attenuated cardiac hypertrophy induced by pressure overload or chronic β-adrenergic stimulation.^96^ In addition to transcription factor regulation, another well-established downstream effector of Akt is mTOR. Multiple studies have demonstrated that rapamycin, a specific mTOR inhibitor, attenuates pathological cardiac hypertrophy.^97,98^

##### Noncoding RNAs

**Fig. 2:**
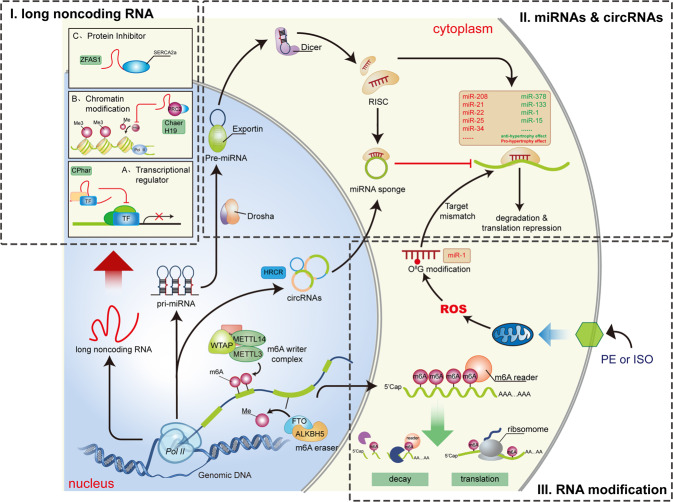
RNA related mechanism of cardiac hypertrophy. Recent studies on RNA creates a novel regulatory network for cardiac hypertrophy. (I) Long non-coding RNAs (lncRNAs) are molecularly multi-functional. They can physically interact with and modulate function of cytoplasmic protein, chromatin remodeling factors, and transcription factors. (II) MicroRNAs (miRNAs) target mRNA and suppressed its expression. Circular RNAs (circRNAs) are also multifunctional, but cardiac hypertrophy related researches mainly focuses on their role as miRNA sponges. (III) RNA modification further adds to the complexity of the regulatory network. N6-methyladenosine (m6A) alters the function of mRNA. Modulating m6A “writer” or “eraser” can affect the development of cardiac remodeling. Oxidative stress creates 8-oxoguanine modification on miR-1, which leads to its target mismatch and triggers hypertrophic response. miRNA: microRNA; circRNA: circular RNA; ZFAS1: Zinc finger antisense 1; PRC2: polycomb repressor complex 2; Chaer: cardiac-hypertrophy-associated epigenetic regulator; SERCA2a: sarco/endoplasmic reticulum Ca2+-ATPase 2a; CPhar: cardiac physiological hypertrophy-associated regulator; TF: transcription factor; RISC: RNA Induced Silencing Complex; HRCR: heart-related circRNA; ROS: reactive oxygen species; WTAP: Wilms’ tumor 1-associating protein; METTL3: methyltransferase like 3; METTL14: methyltransferase like 14; m6A: N6-methyladenosine; FTO: fat mass and obesity associated gene; PE: Phenylephrine; ISO: isoprenaline

With the development of RNA sequencing techniques, many transcripts without coding potential have been identified. Heart failure patients had extensive dysregulation of noncoding RNA in both myocardial tissue and serum.^99,100^ Studies from recent decades have demonstrated the extensive involvement of these noncoding RNAs in the pathophysiology of cardiac hypertrophy (Fig. 2). Noncoding RNAs are categorized according to their length: <200 nucleotides small noncoding RNAs and >200 nucleotides long noncoding RNAs (lncRNAs).

**MicroRNAs**: The most widely studied small noncoding RNAs are microRNAs (miRNAs). These RNAs are single-stranded RNAs of ~22 nucleotides in length that function in RNA silencing. miRNAs bind mRNA molecules, mostly at the 3’ untranslated region (UTR), by base pairing with complementary sequences. The binding of a miRNA leads to translational repression or mRNA degradation. Numerous miRNAs have been shown to regulate cardiac hypertrophy. In vivo inhibition of miR-133 using chemically modified antisense oligonucleotides (ASOs) caused sustained cardiac hypertrophy. The antihypertrophic effect of miR-133 might be mediated by the downregulation of its targets ras homolog family member A (RhoA), cell division cycle 42 (Cdc42), and negative elongation factor complex member A (NELFA).^101^ Another study showed that miR-133 attenuated cardiac hypertrophy and apoptosis induced by pressure overload or β-adrenergic stimulation by targeting adenylate cyclase VI and the downstream cAMP-dependent pathway.^102^ miR-378 repressed cardiac hypertrophy by targeting multiple components in the MAPK pathway: MAPK1, IGF-1 receptor, growth factor receptor-bound protein 2, and kinase suppressor of ras 1.^103^ There are also pro-hypertrophic miRNAs, such as miR-208,^104^ miR-22,^105^ miR-21,^106^ miR-25,^107^ miR-34,^108^ miR-199a,^109^ miR-212/132,^110^ and miR-23.^111^ Interestingly, miR-320 had “double faces” in heart failure. Overexpression of miR-320 in cardiomyocyte deteriorated transverse aortic constriction (TAC) induced cardiac hypertrophy and heart failure, while overexpression in fibroblast attenuated these phenotypes. These opposite effects might be due to different targets of miR-320 in those two cell types. In cardiomyocytes, miR-320 targeted an anti-hypertrophic protein pleckstrin homology domain containing M3, while in fibroblasts it targeted interferon induced transmembrane protein 1.^112^ Targeting pro-hypertrophic miRNAs in vivo using chemically modified inhibitory oligonucleotides showed promising therapeutic effects. However, the delivery of the oligonucleotides is mostly systematic. A cardiac-specific delivery system would further improve the translational potential of this strategy. Ultrasound-targeted microbubble cavitation (UTMC) was proposed to solve this problem. Modified antimiR-23a loaded on cationic lipid-coated microbubbles was infused through a jugular cannula. Ultrasound directed at the heart destroyed the microbubbles and promoted antimiR-23a release in the heart. This approach was effective in preventing cardiac hypertrophy induced by PE .^113^

**LncRNAs**: Multiple lncRNAs are also involved in cardiac hypertrophy. Unlike miRNAs, lncRNAs have various molecular functions in cardiomyocytes. Myosin heavy chain-associated RNA transcripts (Mhrts) are a cluster of antisense transcripts at the Myh7 gene locus. Mhrts are downregulated in pressure overload-induced cardiac hypertrophy. Restoration of the expression of one Mhrt (Mhrt799) improved cardiac function and reduced hypertrophy after TAC surgery. Mechanistically, Mhrt and Brg1 formed a feedback loop to regulate hypertrophy. Brg1 is a chromatin remodeling factor that promotes hypertrophy-related transcriptomic changes. Under stress, Brg1, which is a component of the chromatin repressor, suppressed the transcription of Mhrt. Then, Mhrt bound to the helicase domain of Brg1, sequestering it from its genomic DNA targets and preventing subsequent chromatin remodeling.^114^ Cardiac hypertrophy-associated epigenetic regulator (Chaer) is a heart-enriched hypertrophic lncRNA. Genetic deletion of Chaer attenuated TAC-induced cardiac hypertrophy. Chaer interacts with the catalytic subunit of polycomb repressor complex 2 (PRC2), preventing histone H3 lysine 27 (H3K27) methylation at the target DNA and inducing hypertrophic gene. The interaction of Chaer and PRC2 is induced by stress and is necessary for epigenetic reprogramming in hypertrophy. Interestingly, inhibiting Chaer before but not after TAC efficiently suppressed cardiac hypertrophy, which suggested its role in the initiation of hypertrophy.^115^ The lncRNA H19 is also enriched in muscle. H19 is upregulated at the early phase of pressure overload-induced cardiac hypertrophy but is downregulated at later stages. H19 repression was also observed in a pig model of cardiac hypertrophy and diseased human hearts. H19 knockout promotes cardiac hypertrophy, while H19 overexpression inhibits cardiac hypertrophy. H19 also inhibits PRC2 activity through physical interactions and represses H3K27 trimethylation (H3K27me3) at the tescalcin promoter, which reduces the inhibitory effect of tescalcin on NFAT activity.^116^ Interestingly, H19 appeared to function differently in the right ventricle. Plasma H19 was upregulated in pulmonary artery hypertension patients with decompensated right heart failure. Serum H19 levels predicted the prognosis of patients with idiopathic pulmonary artery hypertension. Silencing H19 protected the right ventricle from hypertrophy and fibrosis in animal models of pulmonary artery hypertension.^117^ In addition to the abovementioned studies, there is much evidence that lncRNAs modulate cardiac function in other disease models. For example, the lncRNA ZFAS1 acts as an inhibitor of sarco/endoplasmic reticulum Ca^2+^-ATPase 2a (SERCA2a) and causes contractile dysfunction in myocardial infarction.^118^

Unlike pathological hypertrophy induced by stress, exercise-induced cardiac hypertrophy is considered physiological and beneficial. Understanding the molecular mechanism of physiological hypertrophy may contribute to cardiovascular disease prevention. The lncRNA cardiac physiological hypertrophy-associated regulator (CPhar) is upregulated during swimming training. CPhar was shown to be necessary for physiological heart growth. Mechanistically, CPhar interacts with DEAD-Box Helicase 17 (DDX17) and prevents the interaction of CCAAT/enhancer binding protein beta (C/EBPβ) with its DNA targets, which ultimately leads to a decrease in the transcription of ATF7.^119^ Another study induced pathological hypertrophy in mice with physiological hypertrophy preconditioning induced by swimming training compared with untrained mice. Exercise-induced hypertrophy preconditioning was shown to attenuate cardiomyopathy marker increases, pulmonary congestion, and cardiac dysfunction after TAC surgery. Exercise training induced Mhrt779 expression via H3K4me3 and H3K36me3 at the promoter region. Mhrt779 inhibited the activation of the histone deacetylase 2 (HDAC2)/Akt/GSK3β pathway during pathological cardiac hypertrophy.^120^

Circular RNAs (circRNAs) are a special class of noncoding RNAs in which an RNA circle formed by back splicing of linear RNA. Although circRNAs play various molecular roles in different cell types, currently identified cardiac hypertrophy-related circRNAs mainly function as miRNA sponges. For example, heart-related circRNA (HRCR) inhibits cardiac hypertrophy by acting as a miR-233 sponge to limit its activity. As a result, the expression of the miR-223 target activity-regulated cytoskeleton-associated protein is increased and antagonized cardiac hypertrophy.^121^ circRNAs normally lack a structural 5’ cap and 3’ polyA tail and are resistant to degradation mediated by RNases. In addition, one circRNA molecule has multiple miRNA binding sites, which makes it an ideal tool for targeting pathogenic miRNAs.^122^ The miRNA-212/132 family has been suggested to have a hypertrophic effect.^110^ Targeting the miR-212/132 family using ASOs (antagomiR-212/132) was successful in treating heart failure in a pig model.^123^ An engineered circRNA with binding sites for miR-212/132 was developed to target these miRNAs. This engineered circRNA had higher efficacy and stability than antagomiR-212/132. Adeno-associated virus-based overexpression of the engineered circRNA attenuated cardiac hypertrophy induced by pressure overload.^124^

Although the abovementioned RNAs are classified as noncoding RNAs, recent studies have shown that some lncRNAs and circRNAs encode micropeptides. Our group analyzed the translatomes of hypertrophic cardiomyocytes and identified multiple micropeptides encoded by lncRNAs. Several of these micropeptides regulated PE-induced pathological cardiomyocyte hypertrophy in an in vitro model.^125^ Dwarf open reading frame (DWORF) is a micropeptide encoded by a putative muscle-enriched lncRNA transcript. DWORF interacts with SERCA and enhances its activity by sequestering phospholamban (PLN). DWORF overexpression counteracted contractile dysfunction caused by PLN overexpression. In a mouse model of dilated cardiomyopathy, the overexpression of DWORF restored cardiac function and prevented cardiac hypertrophy and Ca^2+^ mishandling.^126^

##### RNA modifications

RNA can be chemically modified. There are more than 100 types of posttranscriptional modifications of RNA.^127^ N6-methyladenosine (m6A) represents the most abundant internal RNA modification. m6A has important roles in RNA stability and translation. During the progression of cardiac hypertrophy and heart failure, the landscape of m6A changes and is tightly associated with translatomic changes independent of RNA levels.^128^ These data indicated that RNA m6A modification could be involved in cardiac hypertrophy. Currently, 2 RNA m6A writers have been identified: methyltransferase-like protein 3 (METTL3) and METTL14. Obesity-associated gene (FTO) and AlkB homolog 5 RNA demethylase (ALKBH5) serve as erasers to maintain the balance of m6A. FTO expression in the hearts of heart failure patients was significantly lower than that in the hearts of control individuals. Restoring FTO expression in heart failure attenuated the ischemia-induced increase in m6A and rescued cardiac contractile function. FTO demethylated cardiac contractile-related RNAs, prevented their degradation, and improved protein expression under ischemic conditions.^129^ METTL3-mediated m6A was also involved in normal cardiac function and the hypertrophic response. METTL3 overexpression was sufficient to promote cardiac hypertrophy. METTL3-knockout mice were more likely to develop heart failure with stress and aging than wild-type mice.^130^ Cardiac hypertrophy-associated piRNA (CHAPIR) can regulate cardiac hypertrophy by modulating m6A. The interaction of CHAPIR with METTL3 blocked m6A in the protein mono-ADP-ribosyltransferase (PARP10) transcript, which resulted in an increase in PARP10 protein levels. PARP10 promoted the mono-ADP-ribosylation of GSK3β and inhibited GSK3β activity. NFATc4 is then released from the inhibitory effect of GSK3β, translocates into the nucleus and drives the expression of hypertrophic genes.^131^ In addition to mRNA modifications, noncoding RNA modifications have also been reported to regulate cardiac hypertrophy. Reactive oxygen species (ROS) can oxidize biomolecules. RNA 8-oxoguanine (o^8^G) is one such modification. Under adrenergic agonist stimuli, o^8^G predominantly occurred at position 7 of miR-1. With oxidative modifications, o^8^G can be base paired with A. Introducing 7o^8^G-miR-1 (miR-1 with o^8^G at position 7) or 7U-miR-1 (miR-1 with a G to U replacement at position 7) was sufficient to induce cardiac hypertrophy.^132^ The discovery of RNA modifications involved in cardiac hypertrophy added to the complexity of the regulatory network of gene expression. However, studies in this field are just at the beginning, and much still remains to be explored.

#### Cardiomyocyte death

**Fig. 3:**
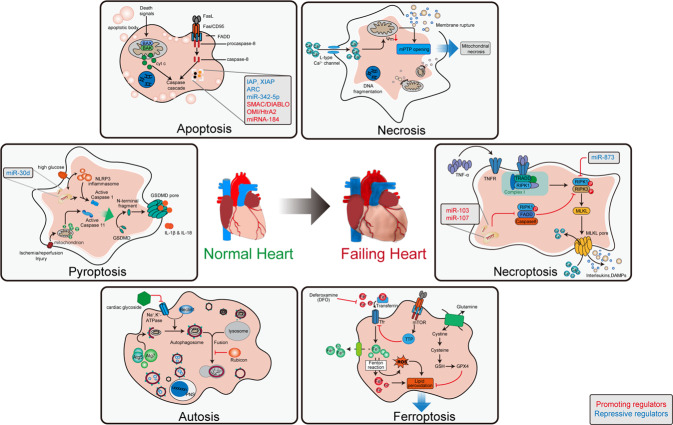
Non-programmed and programmed cardiomyocyte death in failing heart. Cardiomyocyte death contribute to loss of myocardium, especially after ischemia injury. Different types of programmed cell death are controlled by their specific signaling pathway. Modulating programmed cell death by targeting their signaling pathway can attenuate cardiac dysfunction after ischemia injury. BAX: BCL-2-associated X protein; BAK: BCL2-antagonist/killer 1; cty C: Cytochrome C; FADD: Fas associated death domain; FasL: Fas ligand; TNF-α: tumor necrosis factor α; TRAIL: TNF-related apoptosis inducing ligand; IAP, Inhibitor of apoptosis; ARC: Apoptosis repressor with caspase recruitment domain; mPTP: mitochondrial permeability transition pore; TNFR: TNF receptor; TRADD: TNFR1-associated death domain; RIPK1: serine/threonine kinases receptor interacting protein kinase 1; MLKL: mixed lineage kinase-like domain; DAMPs: damage-associated molecular patterns; GSDMD: gasdermin D; PNS: perinuclear space; Atg5: Autophagy related 5; Atg3: Autophagy related 3; Tfr: transferrin receptor; TTP: tristetraprolin; GPX4: glutathione peroxidase 4; GSH: glutathione

Myocardial loss and cardiomyocyte death are distinguishing features of some cardiac diseases, such as myocardial infarction. Progressive cell death ultimately leads to insufficient functional cardiomyocytes and chronic heart failure. In the 20th century, cell death was identified by optical microscopy. When cells are harmed by chemical, physical, or biological insults, they undergo organelle swelling and the loss of cell structure.^133^ Subsequent studies showed that in some instances, cell death is regulated by signaling pathways^134^ and is known as programmed cell death (PCD). After PCD signals stimulate membrane or cytoplasmic proteins, they are transduced through a cascade of protein modifications and trigger apoptosis. There were 3 major features of apoptosis^135^: 1) cell shrinkage, the formation of apoptotic bodies, plasma membrane blebbing, and chromatin condensation; 2) apoptotic body uptake by macrophages; and 3) the integrity of apoptotic bodies and absence of inflammatory and immune responses caused by the leakage of intracellular contents.^136,137^ Recent decades have witnessed the discovery of other forms of PCD: ferroptosis^138^ pyroptosis,^139,140^ autophagy-dependent cell death,^141^ and necroptosis.^142,143^ Both PCD and nonprogrammed cell death have been suggested to be involved in multiple cardiac diseases, including ischemic cardiomyopathy, chronic heart failure, myocarditis, and congenital cardiomyopathy (Fig. 3).^144–148^

##### Apoptosis

Apoptosis can be initiated by the cell surface death receptor pathway or mitochondrial pathway. Both pathways lead to caspase activation. Ligands that activate the cell surface death receptor pathway include Fas ligand (FasL), tumor necrosis factor α (TNF-α), and TNF-related apoptosis inducing ligand (TRAIL). Corresponding receptors are members of the TNF receptor superfamily. The ligand–receptor interaction triggers the formation of the death-inducing signaling complex (DISC) by the Fas-associated death domain (FADD) protein and procaspase-8/10.^149–151^ After procaspase-8 enters the DISC, it is processed into the mature initiator caspase-8. Then, mature caspase-8 catalyzes the conversion of procaspase-3 to effector caspase-3. The mitochondrial pathway is usually activated by intracellular stimuli, such as DNA injury, nutrition deprivation, and oxidative stress.^152^ B cell lymphoma 2 (BCL-2) family members, such as Bax, Bak, Bcl-2, and Bcl-xL, change the permeability of the mitochondrial membrane and regulate the release of apoptotic molecules.^153^ Among these apoptotic molecules, cytochrome C links procaspase-9 to apoptotic protease-activating factor 1 (APAF1) and forms a complex called the apoptosome. The apoptosome activates the initiator caspase-9, which in turn activates the effector caspase-3.^154^

Very early studies concluded that necrosis is the major form of cell death in cardiac ischemia–reperfusion injury. However, a considerable amount of apoptosis was also observed.^147,155^ Multiple studies have shown that inhibiting the cell surface death receptor pathway or mitochondrial pathway reduces cardiomyocyte apoptosis and infarct size in an ischemia–reperfusion (I/R) model. I/R injury stimulates the expression of FasL. Compared with wild-type mice, mice with a loss-of-function mutation in TNF receptor superfamily member 6 had less apoptosis, as measured by terminal deoxynucleotidyl transferase dUTP nick end labeling (TUNEL).^156^ Genetic deletion of the proapoptotic factor Bax or overexpression of the antiapoptotic factor Bcl-2 limited myocardial infarction in an I/R model.^157,158^ Human chronic heart failure samples had more TUNEL-positive apoptotic cells than nonheart failure control samples.^146^ Collectively, these data suggested the involvement of apoptosis in the chronic heart failure.

Apart from these canonical apoptosis pathways, other protein or RNA regulators are involved in cardiomyocyte apoptosis. Inhibitors of apoptosis (IAPs), such as cellular IAP1 (cIAP1), cIAP2, and X-linked IAP (XIAP), inhibit caspase activity and negatively regulate apoptosis.^159^ Cardiac overexpression of cIAP2 reduced the number of TUNEL-positive cells and infarct size after I/R injury.^160^ The apoptosis repressor with caspase recruitment domain (ARC) interferes with the interaction between the DISC and FADD and consequently downregulates the cell surface death receptor apoptosis pathway.^161^ In addition, ARC can also inhibit the conformational change in Bax into an active form and inhibit the mitochondrial pathway. Under I/R injury, ARC undergoes ubiquitin-mediated proteasomal degradation. Cardiac-specific overexpression of ARC protected cardiomyocytes from I/R injury and hypoxia-induced cell death.^161,162^ AMPKα2 protected against cardiomyocyte apoptosis in a pressure-overload induced heart failure model. AMPKα2 interacted with PTEN-induced putative kinase 1 (PINK1) and enhanced the activity of PINK1-Parkin-sequestosome-1 pathway involved in cardiac mitophagy, which led to elimination of damaged mitochondria, improvement in mitochondrial function, decrease in reactive oxygen species production, and apoptosis of cardiomyocytes.^163^ miR-342-5p targeted Caspase 9 and JNK2 to inhibit I/R-induced cardiomyocyte apoptosis.^164^ miR-320 promoted cardiomyocyte apoptosis and exacerbated cardiac function in a model of diabetic cardiomyopathy. Mechanistically, miR-320 recognized promoter region of CD36 (a fatty acid translocase) and recruited argonaute RISC catalytic component 2 (Ago2) to the promoter, which resulted in enhanced CD36 transcription. Increased CD36 protein induced cardiomyocyte apoptosis by promoting lipotoxicity.^165^ I/R stress stimulates ROS generation, which can cause oxidative modifications of specific molecules and lead to dysfunction. Bcl-xL and Bcl-w are not natural target of miR-184. However, oxidized miR-184 was associated with the 3’ UTRs of Bcl-xL and Bcl-w, which led to the downregulation of these proteins and the initiation of apoptosis in cardiomyocytes.^166^

##### Necroptosis

Necroptosis is another form of PCD with features of both necrosis and apoptosis. Like necrotic cell death, necroptosis is also characterized by the leakage of intracellular contents, which occurs in a tightly regulated manner.^167^ When TNF receptors receive corresponding stimuli, homologous receptor interacting protein kinase 1 (RIPK1) is recruited. RIPK1 and receptor interacting serine/threonine kinase 3 (RIPK3) phosphorylate each other and form a complex called the necrosome, which in turn phosphorylates and activates mixed lineage kinase-like domain (MLKL). Activated MLKL forms oligomers, which are inserted into the cell membrane. As a result, membrane permeability changes, and necroptosis is initiated.^168^ Administration of the RIPK1 inhibitor necrostatin-1 was able to reduce infarct size in an I/R model.^169,170^ RIPK3 and MLKL deficiency protected tissue from I/R injury, but RIPK3 provided greater protection than MLKL.^171^ Later studies showed that RIPK3 was not only involved in the classic RIPK1-RIPK3-MLKL necroptosis pathway but could also induce necroptosis by activating CamK II and triggering opening of the mitochondrial permeability transition pore (mPTP). The RIPK3-CamK II-mPTP pathway is involved in both I/R-related and doxorubicin-induced cardiomyocyte necroptosis and heart failure.^172^ As mentioned previously, FADD is an important regulator of apoptosis and has been shown to regulate programmed necrosis. FADD knockout promoted H_2_O_2_-induced cardiomyocyte necroptosis but inhibited apoptosis. FADD negatively regulates necroptosis by inhibiting the interaction of RIPK1 and RIPK3. miRNAs targeting FADD promote necroptosis and I/R-induced myocardial injury.^173^ In contrast, miR-873, which targets RIPK1/RIPK3, downregulated necroptosis and reduced infarct size in the I/R model. The lncRNA necrosis-related factor (NRF) acts as an endogenous sponge of miR-873 and therefore upregulates necroptosis and myocardial I/R injury.^174^

##### Pyroptosis

Pyroptosis was discovered early in 1992 by observing the rapid cytolysis of macrophages infected with *Shigella flexneri*. Pyroptosis is a highly inflammatory form of PCD. Pyroptosis signaling triggers the formation of a multiprotein complex called the inflammasome, which activates a different set of caspases than apoptosis. Activated caspase-1 cleaves gasdermin D (GSDMD), producing the functional GSDMD N-terminal domain (GSDMD-N). GSDMD-N can oligomerize and form pores in the cell membrane, which allows the leakage of intracellular contents, including IL-1β and IL-18, water influx, and cell swelling and bursting. The family member GSDME is cleaved by caspase-3 and is also involved in pyroptosis.^175,176^ To date, pyroptosis has been observed in various cell types, including monocytes, macrophages, dendritic cells, cardiomyocytes, and fibroblasts.^177^ Because the inflammatory response is involved in I/R injury, pyroptosis may also play a role. A large number of inflammasomes can be observed in cardiac I/R injury. Germline deletion of the pyroptosis-related genes apoptosis-associated speck-like adapter protein (ASC) and caspase-1 protected the heart from I/R injury.^178^ However, as there are many cell types in the heart, the specific role of cardiomyocyte pyroptosis must be demonstrated by a study with cell type-specific genetic modification. Indeed, deletion of GSDMD in cardiomyocytes had a similar protective effect. Interestingly, studies showed that cardiomyocyte pyroptosis was mediated by caspase-11 but not classic caspase-1.^179^ In diabetic cardiomyopathy, miR-30d targets forkhead box O3 (FOXO3a), which in turn leads to a decrease in ARC, caspase-1 activation, and pyroptosis.^180^ Activated NLR family pyrin domain containing 3 (NLRP3) inflammasomes and cardiomyocyte pyroptosis can be observed in myocardial tissues from dilated cardiomyopathy patients. Doxorubicin-induced dilated cardiomyopathy was also associated with NLRP3 inflammasome-mediated pyroptosis. NLRP3 or caspase-1 deletion can reduce doxorubicin-induced pyroptosis and improve cardiac function.^181^

##### Autosis

Autophagy is a conserved catabolic process that degrades macromolecules (such as proteins, lipids, and nucleic acids) and injured organelles (especially mitochondria).^141^ The target material is first loaded into autophagosomes that are surrounded by a lipid bilayer. The cargo-loaded autophagosomes then merge with lysosomes, where the contents are degraded by acid hydrolase. As autophagy is a self-digestive process and the cell membrane is intact, it does not induce an inflammatory response. Autophagy is a cell survival strategy that responds to stress, but it can also be overactivated in certain circumstances and lead to unnecessary cell death. There are multiple forms of autophagy: macroautophagy, chaperone-mediated autophagy, microautophagy and endosomal microautophagy.^182^ This review mainly focuses on macroautophagy, as this is believed to be the major form in the myocardium. Autophagy is increased in heart failure induced by pressure overload or desmin-related cardiomyopathy.^183,184^ Autophagy related 5 (ATG5) is required for autophagy. Cardiomyocyte-specific deletion of ATG5 in adult mice led to the rapid deterioration of cardiac function under baseline and stress conditions, indicating that constitutive cardiomyocyte autophagy was important for maintaining normal cardiac function and that increased autophagy was an adaptive response to stress.^185^ However, data on Beclin 1, a protein that is required for autophagosome formation, appeared to be conflicting. Heterozygous deletion of Beclin1 decreased cardiomyocyte autophagy and protected the heart from pathological remodeling induced by pressure overload. Beclin 1 overexpression increased both autophagy and cardiac remodeling under stress.^184^ These inconsistent results suggested that autophagy was mediated by different pathways and might have different functional implications. The effect of cardiomyocyte autophagy also differed under different stress conditions. Chemical inhibition of autophagy worsened cardiomyocyte survival in glucose deprivation, which is a condition that mimics myocardial ischemia. However, the downregulation of autophagy by heterozygous Beclin 1 deficiency protected the heart from I/R injury. A mechanistic study showed that ischemia-induced autophagy was AMPK-dependent, while I/R-induced autophagy was not.^186^ These data further emphasized the complexity of the autophagy regulatory network under different conditions. Interestingly, apoptosis was also reduced in Beclin 1^+/−^ mice, as measured by the TUNEL assay.^186^ Further study showed that Beclin 1 could interact with Bcl-xL,^187^ which regulates Bax activity and apoptosis.^188^ This inconsistency was also present in diabetic cardiomyopathy. Inhibiting autophagy by downregulating Beclin 1 or ATG16 protected the heart in diabetic mice,^189^ but another study indicated that the benefit of metformin was mediated by the upregulation of autophagy in the heart.^190^ It is worth pointing out that autophagy is not always the direct driving force of cell death. Instead, autophagy can accompany cell death or be triggered by cell death.^191^

Autosis, a form of autophagy-dependent cell death, has been proposed recently. Autosis can be induced by autophagy-inducing peptides, starvation, and hypoxia-ischemia, where Na^+^, K^+^-ATPase plays an important role. Cardiac glycosides or knockdown of the Na^+^, K^+^-ATPase α1 subunit can effectively inhibit autosis.^192^ Autosis morphologically differs from apoptosis and necrosis. Autosis is characterized by autophagic bodies and dilation and fragmentation of the endoplasmic reticulum at an early stage, swollen perinuclear spaces, electron-dense mitochondria, an empty ballooning space with the membrane starting to merge with the outer nuclear membrane, focal nuclear concavity at the following stage, and empty focal ballooning perinuclear spaces and a marked decrease in cytoplasmic organelles at the final stage. More importantly, autosis can only be rescued by autophagy inhibitors but not inhibitors of other forms of cell death.^193^ During cardiac I/R injury, the upregulation of Rubicon resulted in the accumulation of autophagosomes and increased autosis. Inhibition of autosis either by inhibiting Rubicon or administering cardiac glycoside reduced I/R injury.^194^

The functional implication of autophagy-dependent cell death varies significantly in different pathological processes and even at different stages in the same pathological process. Therefore, much remains to be studied before considering the therapeutic potential of targeting autophagy.

##### Ferroptosis

Ferroptosis is a form of PCD that is highly dependent on intracellular iron and is characterized by lipid peroxidation damage. Dense, compact mitochondria with loss of cristae are distinct morphological characteristics.^195^ ROS generation caused by excess intracellular iron accumulation is the main driver of ferroptosis. When the amount of iron exceeds the intracellular storage capacity, ferrous iron in the liable iron pool (LIP) increases and reacts with peroxide to generate ROS, which is known as the Fenton reaction. ROS attack lipids on the membrane and generate toxic lipid peroxides. The antioxidant system is the major negative regulator of ferroptosis, among which glutathione peroxidase 4 (GPX-4) plays a fundamental role. GPX-4 and glutathione (GSH) convert toxic lipid hydroperoxides to nontoxic lipid alcohols. In cardiac I/R injury, abundant iron and oxygen in the blood provide ideal substrates for the Fenton reaction. Moreover, I/R downregulates the antioxidant system, predisposing cells to ferroptosis. A cardiac magnetic resonance imaging study showed that residual myocardial iron caused by intramyocardial hemorrhage in patients with myocardial infarction was associated with left ventricular remodeling during follow-up.^196^ In a cross-sectional study on chronic stable angina patients, serum iron was positively associated with the severity of coronary heart disease.^197^ In a study of isolated perfused mouse hearts, ferroptosis was found to be involved in I/R injury. The iron chelator deferoxamine (DFO) or ferroptosis inhibitor ferrostatin-1 successfully protected the heart from I/R injury.^198^ In doxorubicin-induced cardiomyopathy, nuclear factor erythroid 2-related factor 2 (NRF2) translocates to the nucleus and upregulates the expression of heme oxygenase 1 (HMOX1), which degrades heme and releases free iron in cardiomyocytes and induces ferroptosis. Zinc protoporphyrin IX, an HMOX1 antagonist, attenuated cardiac injury induced by doxorubicin. Interestingly, excess free iron accumulated mainly in mitochondria and caused lipid peroxidation on the membrane. Compared with nontargeted antioxidants, mitochondria-targeted antioxidants had a more robust cardioprotective effect.^199^ mTOR is also involved in the regulation of cardiomyocyte ferroptosis. Rapamycin, an mTOR inhibitor, rescued cardiomyocyte death triggered by ferroptosis inducers.^200^ This outcome could be explained by the role of mTOR in iron hemostasis. mTOR can modulate transferrin receptor 1 stability and alter cellular iron flux through tristetraprolin.^201^

### Fibroblasts

Chronic heart failure is often accompanied by cardiac fibrosis. Cardiac fibrosis manifests as the deposition of collagen and other extracellular matrix (ECM) components. The most important step is the activation of cardiac fibroblasts and their transformation into myofibroblasts. Under stress, cardiac fibrosis maintains the integrity of the heart when functional cardiomyocytes are lost, but prolonged fibrosis is pathologic, leading to cardiac stiffness and diastolic dysfunction.^202,203^ Cardiac fibrosis is not just a matter of cardiac fibroblasts. Instead, this process involves various intracellular interactions and crosstalk, especially in the injured heart.

#### Transforming growth factor β (TGF-β) signaling

TGF-β is the most widely studied cytokine associated with fibrosis in multiple organs.^204–207^ In the mammalian heart, three isoforms, TGF-β1, -β2 and -β3, constitute the TGF-β family.^208^ TGF-β1 is widely distributed in tissues and appears to be the strongest regulator of fibrosis.^209^ TGF-β and its binding proteins are secreted as a latent complex and are located in the ECM. Multiple activation steps are required to release mature TGF-β.^210^ The latent TGF-β complex serves as a sensor that can be activated by signals from multiple activators, such as proteases, integrin and ROS.^211,212^ In mammals, there are 7 type I receptors (TβRI, also known as activin-like receptor kinase (ALK) 1–7) and 5 type II receptors (TβRII), which are all transmembrane serine/threonine kinases.^213^ The binding of TGF-β to TβRII induces the formation of a heterotetrameric complex by two TβRI and two TβRII units, which phosphorylate and activate the cytoplasmic domain of TβRI and trigger a downstream cascade.^214,215^

The Smad-dependent TGF-β signaling pathway in fibrosis is the most established. TβRI is activated by TGF-β and can bind and phosphorylate intracellular receptor-activated Smads (R-Smads). Specifically, ALK5 activates Smad2 and Smad3, while ALK1 and ALK2 activate Smad1, Smad5, and Smad8. Subsequently, the activated R-Smads form complexes with Smads in the cytoplasm, which then translocate to the nucleus and modulate gene expression as transcription factors.^216–218^ The TGF-β/ALK5/Smad2/3 axis is suggested to play a dominant role in cardiac fibrosis. Other cascades, such as the ALK1/Smad1 axis^219^ and Smad7-dependent signaling,^220,221^ are also involved in cardiac fibrosis, which, however, is less well established.

Mice with Smad3 global deficiency had reduced collagen deposition and inflammatory infiltration after myocardial infarction. Although the infarct size was not changed, dilative remodeling and diastolic dysfunction were attenuated.^222^ However, TGF-β1/Smad3 signaling plays different roles in fibroblasts and cardiomyocytes. Fibroblast-specific deletion of Smad3 paradoxically led to unrestrained fibroblast proliferation, impaired scar remodeling, reduced collagen synthesis, and perturbed alignment of myofibroblasts after myocardial infarction by suppressing integrin-nicotinamide adenine dinucleotide phosphate oxidase-2 (NOX-2) signaling. As a consequence, adverse cardiac remodeling was accentuated. In contrast, cardiomyocyte-specific loss of Smad3 attenuated remodeling and reduced cardiomyocyte apoptosis, which was associated with decreased NOX-2, nitrosative stress, and matrix metalloproteinase 2 (MMP-2) levels in the myocardium.^223^ In a cardiac hypertrophy model induced by pressure overload, however, Smad3 deletion worsened cardiac hypertrophy and survival after TAC surgery, although it did reduce cardiac fibrosis.^224^ Myofibroblast-specific Smad3-knockout mice also showed unexpectedly exacerbated systolic dysfunction after TAC surgery. Phosphorylated Smad3 inhibited the expression of MMP-3 and MMP-8 and upregulated the expression of tissue inhibitor of metalloproteinases-1 (TIMP-1), which reduced ECM fragmentation, decreased inflammation driven by macrophages, and ultimately protected cardiomyocytes from necrosis and dysfunction.^225^ In an obese diabetic mouse model, heterozygous Smad3 deletion resulted in less fibrosis, but Smad3-null mice exhibited early lethality.^226^ Altogether, Smad3-mediated fibrosis plays dual roles in the heart. Smad3 is necessary for the adaptive fibroblast response to cardiac stress but also contributes to maladaptive cardiac fibrosis. The role of Smad2 in cardiac fibrosis is less clear. In vitro knockdown of Smad2 reduced collagen V expression and fibronectin, periostin, and versican synthesis in cultured cardiac fibroblasts.^227^ However, fibroblast- or myofibroblast-specific Smad2-knockout mice^227–229^ had a normal cardiac phenotype and function, without changes in fibrosis induced by pressure overload. In addition to these classic factors in the cascade, multiple players involved in the TGF-β/ALK5/Smad2/3 signaling pathway also modulate cardiac fibrosis.

##### Regulators of cardiac fibroblasts

Vascular peroxidase 1 (VPO1) is a heme enzyme that transforms hydrogen peroxide (H_2_O_2_) into hypochlorous acid (HClO) and is upregulated and activated in myocardial infarction or by TGF-β stimulation. In vivo and in vitro loss-of-function studies showed that VPO1 promoted cardiac fibroblast proliferation, migration and differentiation by catalyzing HClO formation, which further activated downstream Smad2/3.^230^ In a pressure overload model, Sirtuin 1 activation ameliorated cardiac fibrosis and hypertrophy by reducing the transcriptional activity of Smad2/3 by decreasing the acetylation level.^231^ In a myocardial fibrosis model induced by a combination of angiotensin II infusion and cardiac pressure overload, TIMP-1 exacerbated myocardial fibrosis in a metalloproteinase-independent manner. Specifically, TIMP1 induced the interaction between CD63 (a cell surface receptor of TIMP1) and integrin-β1 in fibroblasts, which initiated the activation and nuclear translocation of Smad2/3 and β-catenin and subsequent collagen synthesis.^232^ Moreover, periostin, an ECM protein that is mainly secreted by osteoblasts and fibroblasts, was upregulated in human samples of chronic heart failure and angiotensin II-induced heart failure. This upregulation was partly dependent on TGF-β1/Smad signaling.^233^ Recently, Chen et al.^234^ identified WW domain containing E3 ubiquitin protein ligase 2 (WWP2) as a regulator of the profibrotic gene network in diseased rat and human hearts. Specifically, in primary cardiac fibroblasts, the WWP2 N-terminal isoform translocated into the nucleus in response to TGF-β1 stimulation. WWP2 subsequently bound to Smad2 in the nucleus and initiated the transcription of ECM-related genes. Intriguingly, a type of primary cilium in neonatal and adult cardiac fibroblasts was also found to contribute to fibrogenesis. The primary cilium and its requisite signaling protein PC1 can mediate the activation of the TGF-β1/SMAD3 signaling pathway.^235^

##### Regulators of cardiomyocytes

Although the effect of hypoxia on cardiomyocytes is well understood, hypoxia can also affect fibroblasts by changing the cardiomyocyte secretome. A recent study used a novel mass spectrometry–based secretome analysis of hypoxic cardiomyocytes. Proprotein convertase subtilisin/kexin type 6 (PCSK6) was the most strongly upregulated factor. PCSK6 was secreted into the extracellular space and activated TGF-β, which initiated Smad signaling and ultimately exacerbated cardiac fibrosis.^236^ In addition, in a dilated cardiomyopathy (DCM) mouse model generated by cardiac-specific knockdown of lamin A/C, Yin Yang 1 (YY1) increased the expression of bone morphogenetic protein 7 (BMP7) but suppressed connective tissue growth factor (CTGF) expression in cardiomyocytes, which suppressed TGFβ/Smad signaling in the heart and inhibited fibrosis.^237^

##### Regulators of immune cells

Liu et al.^238^ showed that the eosinophil (EOS)-derived cationic protein mEar1 could inhibit the activation of cardiac fibroblasts by blocking TGF-β-induced Smad2/3 signaling in mice after myocardial infarction. In addition, CD1d, a glycoprotein expressed on antigen-presenting cells and that is recognized by natural killer T (NKT) cells, was suggested to be involved in cardiac remodeling and fibrosis. In an Ang II infusion model, CD1d deletion led to exacerbated fibrosis and inflammation, which was associated with TGF-β1/Smad2/3 pathway activation.^239^

##### Noncoding RNA regulators

The combination of MI and chronic intermittent hypoxia (CIH) exposure increased the expression of miR-214-3p. As a result, one of its targets, cardiac C1q tumor necrosis factor-related protein 9 (CTRP9), was downregulated. The suppression of the cardiokine CTRP9 was responsible for TGF-β/Smad activation and subsequent enhancement of cardiac fibrosis.^240^ miR-29b downregulated TGF-β/Smad3 signaling by directly targeting the coding sequence of TGF-β1, which inhibited cardiac fibrosis induced by Ang II infusion.^241^ In addition, the intracellular transfer of miRNAs is also involved in fibrosis regulation. miR-30d, which is released from cardiomyocytes in extracellular vesicles (EVs), targets integrin subunit alpha 5 (ITGA5) and decreases its expression in cardiac fibroblasts in a paracrine manner, leading to the inhibition of TGF-β1/Smad2/3 signaling and cardiac fibrosis.^242^

##### Crosstalk between TGF-b signaling and MAPK pathway

Interleukin 11 (IL-11) was robustly upregulated in response to TGFβ1 stimulation and mediated its profibrotic effect. Mechanistically, IL-11 promoted fibrosis-associated protein synthesis through ERK signaling in an autocrine manner.^243^ In ischemic injury, myofibroblast- or resident fibroblast-specific MAPK p38α deletion alleviated cardiac fibrosis, while activating p38 signaling in cardiac fibroblasts induced fibrotic remodeling.^244^ However, another study showed that cardiac fibroblast p38α signaling promoted pathological cardiomyocyte hypertrophy through IL-6 paracrine signaling.^245^ Follistatin‐like 1 (FSTL1), a secretory protein, was significantly increased in fibroblasts and myofibroblasts in the infarcted area and after TGF-β1 stimulation. FSTL1 activated fibroblasts through ERK1/2 signaling instead of Smad2/3 signaling. FSTL 1-mediated fibrosis protected the heart from rupture after myocardial infarction.^246^

##### Crosstalk between TGF-b signaling and Wnt pathway

Cardiac fibrosis mediated by TGF-β signaling plays a pivotal role in autoimmune myocarditis. Specifically, the TGF-β/TAK1 cascade triggers Wnt protein secretion and subsequently activated signal transduction, which promotes myofibroblast differentiation and fibrosis progression.^247^ In addition, a recent study showed that miRNAs also regulated crosstalk between Wnt and TGF-β signaling.^248^ Profibrotic stimuli decreased the expression of miR-384-5p in cardiac fibroblasts, which enhanced the expression of a series of receptors [Frizzled class receptor 1 (FZD1), FZD2, LDL receptor related protein 6 (LRP6), and TGF-β receptor type 1 (TGFBR1)]. Subsequently, the TGF-β-induced expression of Wnt3a was increased, which in turn increased TGF-β synthesis, thus forming a TGF-β/Wnt transactivation loop and triggering myofibroblast activation.

#### Wnt signaling

The Wnt1 gene was first identified in breast tumors in 1982,^249^ and the relationship between diseases and the Wnt signaling pathway was described for the first time in 1991.^250,251^ Wnts are a group of secretory proteins and can be found in various species and multiple organs. In vertebrates, 19 Wnt genes have been identified as ligands that can interact with at least 15 receptors to transduce signals.^252^ There are 3 pathways involved in Wnt signaling: 1) the canonical β-catenin-dependent pathway, 2) planar cell polarity, and 3) the calcium pathway.^253^ β-catenin is the most important downstream protein in the canonical pathway, which is the best characterized of the three. In the absence of a Wnt ligand, a complex of assembled proteins consisting of Dishevelled (DVL), Axin, adenomatosis polyposis coli (APC), GSK3, Casein kinase 1 (CK1), protein phosphatase 2 A and the E3-ubiquitin ligase β-TrCP mediate the phosphorylation and ubiquitination of β-catenin, which is subsequently degraded by proteasomes. When extracellular Wnt binds to a heterodimeric receptor complex formed by one FZD and one LRP5/6, FZD recruits DVL, which further triggers Axin recruitment and LRP5/6 phosphorylation by GSK3 and CK1α. Then, the destruction complex for β-catenin is detached, which leads to β-catenin accumulation and nuclear translocation. In the nucleus, β-catenin and T-cell factor (TCF) activate Wnt-responsive genes.^254,255^

In Wnt/planar cell polarity (PCP) signaling, Wnt binds to FZD receptors and induces the serial activation of DVL and Dishevelled associated activator of morphogenesis (DAAM)/Ras homolog family member A (RHOA)/Rho associated coiled-coil containing protein kinase (ROCK) and Rac family small GTPase 1 (RAC1)/JNK, which leads to remodeling of the actin cytoskeleton and cell polarity, respectively.^256,257^ Another β-catenin-independent signaling pathway is Wnt/calcium signaling. Wnt activates FZD receptors and heterotrimeric G proteins, which induce the activation of PLC. Activated PLC catalyzes the formation of IP3 and DAG. IP3 promotes Ca^2+^ release from the endoplasmic reticulum, and Ca^2+^ levels rapidly increase in the cytoplasm. Activated CamKII and calcineurin in turn activate the transcription factors NF-κB and NFAT, respectively. In addition, DAG activates PKC, and PKC in turn activates NF-κB and CREB, which regulate gene expression in a similar way as IP3.^258,259^ The Wnt signaling pathway has been shown to contribute to fibrosis in multiple organs, including the heart.^252^

##### Wnt/β-catenin signaling

An in vitro study showed that Wnt ligands can be transferred to cardiac fibroblasts by extracellular vesicles and activate the Wnt/β-catenin pathway.^260^ Moreover, Wnt1 expression was stimulated by acute cardiac ischemic injury. Wnt1 stimulated cardiac fibroblasts to proliferate and express profibrotic genes. Disruption of Wnt/β-catenin in cardiac fibroblasts negatively affected wound healing and cardiac function after acute ischemic injury.^261^ Wnt10b is expressed by cardiomyocytes and enriched in intercalated discs (IDs). In a model of myocardial infarction, Wnt10b was upregulated in cardiomyocytes at the border zone. Cardiomyocyte-specific overexpression of Wnt10b activated canonical Wnt/β-catenin signaling in endothelial cells and enhanced their proliferation, which triggered neovascularization after heart injury, attenuated fibrosis, and improved heart function.^262^ The Wnt coreceptor LRP6 was shown to have a protective effect on cardiac fibrosis in a recent study. Mechanistically, under pressure overload, cardiomyocyte-specific overexpression of LRP6 enhanced the interaction of LRP6 with cathepsin D (CTSD), which caused the degradation of Wnt5a and Wnt11. As a result, cardiomyocyte-secreted Wnt5a/Wnt11 was reduced, and cardiac fibrosis was eventually attenuated.^263^ In the pressure overload model, Wnt/β-catenin signaling was activated. Cardiac fibroblast-specific loss of β-catenin preserved heart function and alleviated cardiac hypertrophy and fibrosis under TAC by downregulating the expression of collagen genes downstream of TGF-β signaling.^264^ Similar to Smad-mediated fibrosis, Wnt-mediated fibrosis also plays dual roles in different conditions.

**MicroRNA regulators of the Wnt/β-catenin signaling pathway**: miR-27b-3p levels were significantly reduced in the peripheral blood of atrial fibrillation (AF) patients. In this study, miR-27b-3p directly targeted Wnt3a and decreased its expression, which inhibited Wnt/β-catenin signaling and attenuated atrial fibrosis.^265^ Desmoglein-2 (DSG2) is one of the most common pathogenically mutated genes associated with arrhythmogenic cardiomyopathy (AC). Cardiomyocyte-specific overexpression of the human DSG2 mutant in mice triggered AC pathogenesis, including cardiomyocyte necrosis and ventricle fibrosis. miR-708-5p, miR-217-5p and miR-499-5p were significantly dysregulated in this AC model. These dysregulated miRNAs were predicted to regulate Wnt/β-catenin signaling.^266^ miR-29 has been shown to inhibit fibrosis in multiple organs.^267,268^ Global miR-29 knockout, antimiR-29 administration, and cardiomyocyte-specific miR-29 loss all prevented cardiac hypertrophy and fibrosis induced by pressure overload. A mechanistic study suggested that miR-29 repressed Wnt signaling by targeting and inhibiting four factors involved in Wnt signaling [GSK-3β, β-catenin interacting protein 1 (CTNNBIP1), HMG-Box Transcription Factor 1 (HBP1) and GLIS Family Zinc Finger 2 (GLIS2)] in cardiomyocytes. The activation of Wnt signaling due to miR-29 loss triggered cardiac hypertrophy and the secretion of profibrotic factors, which could stimulate cardiac fibroblasts.^269^

**Regulatory role of secreted Frizzled-related proteins (sFRPs) in Wnt/β-catenin signaling**: sFRPs are normally known as negative regulators of the Wnt signaling pathway and are involved in cardiac fibrosis. The sFRP-1 expression level was increased in samples from chronic heart failure patients. Cardiac fibroblasts lacking sFRP-1 had increased α-smooth muscle actin expression, cell proliferation rates, and collagen production. Aged sFRP-1 knockout mice spontaneously developed massive cardiac fibrosis and heart failure. The loss of sFRP-1 led to increased expression of Wnt1, Wnt3, Wnt7b, and Wnt16 and Wnt responsive genes, such as Wnt1 inducible signaling pathway protein 1 (WISP1) and lymphoid enhancer binding factor 1 (LEF1), along with increased β-catenin protein levels.^270^ sFRP-4 was also reported to play a cardioprotective role.^271^ In contrast to sFRP-1 and sFRP-4, there was evidence suggesting that sFRP2 could activate Wnt/β-catenin signaling and promote proliferation in cardiac fibroblasts.^272^

##### Wnt/calcium signaling pathway

CaMKII is a key downstream effector in the Wnt/calcium pathway^273^ and is involved in cardiac remodeling. Wnt signaling activation by transgenic overexpression of DVL-1 led to severe heart failure accompanied by cardiac hypertrophy and fibrosis. All 3 downstream Wnt pathways were activated in transgenic mice.^274^ The cardiac phenotype of DVL-1-overexpressing mice was normalized when CaMKIIδγ was deleted. The loss of CaMKII had no impact on Dvl-1-induced activation of β-catenin or JNK, indicating that CaMKII was the major effector of Dvl-1 overexpression-induced cardiomyopathy. Mechanistically, CaMKII coupled Wnt signals to HDAC4 and the activity of MEF2.^275^ However, global genetic modification could not distinguish the role of CaMKII in different cell types in the heart.

### Immune cells

Immune cells, including myeloid and lymphoid cells, account for 10.4% of the total cell population in atria and 5.3% in ventricular tissues.^276^ Numerous types of immune cells, including macrophages, neutrophils, eosinophils, dendritic cells (DCs), mast cells (MCs), T cells, B cells, and NK cells, can be found in the heart.^277^ These immune cells have close interactions with other cell types in the heart and play important roles in maintaining cardiac homeostasis.^277^ Interleukin-1β (IL-1β) is secreted by various immune cells and had a pro-inflammatory effect. In CANTOS trial, an IL-1β monoclonal antibody, canakinumab, significantly reduced recurrent cardiovascular events among patients with previous myocardial infarction and a high-sensitivity C-reactive protein level.^278^ What’s more, a subsequent analysis showed that canakinumab treatment also reduced risk of heart failure among these patients.^279^ These data demonstrated the importance of inflammation mediated by immune cells in cardiac remodeling after MI.

Macrophages can be classified as bone marrow-derived macrophages and resident macrophages (rMacs) according to their origins. These cells are engaged in dead cardiomyocyte clearance, the regulation of cardiomyocyte regeneration and left ventricle remodeling.^280^ Macrophages are necessary for neonatal heart regeneration after injury (before P7) but contribute to fibrosis and final scar formation in the adult heart.^281^ Macrophages differentiate into 2 subgroups, namely, M1 or M2, in response to pro- or anti-inflammatory signals. Proinflammatory M1 macrophages have enhanced phagocytosis and antigen presentation, while reparative M2 macrophages are characterized by increased secretion of anti-inflammatory cytokines.^282^ M1 macrophages have been shown to have a protective role in the infarcted heart by antagonizing cardiomyocyte apoptosis, while M2 macrophages enhance cardiac fibrosis and angiogenesis to promote cardiac repair.^283^ Neutrophils play a reparative role by accelerating the growth of endothelial cells and angiogenesis during myocardial infarction but attenuate cardiac hypertrophy by reducing macrophage activation.^284^ Horckmans et al. constructed a neutrophil depleted mice via intraperitoneal injection of monoclonal antibody clone 1A8. These mice had more apoptotic cells and worsened cardiac function after MI. Mechanistically, neutrophils secreted gelatinase-associated lipocalin (NGAL) to promote macrophage polarization towards M2c, which was required for apoptotic cell clearance.^285^ DC depleted mice had deteriorated cardiac function and remodeling after MI. These mice had marked infiltration of proinflammatory monocytes and macrophages, but impaired recruitment of anti-inflammatory monocytes and macrophages^286^ MCs are regarded as sentinels in the heart that secrete various proinflammatory and immunoregulatory factors that regulate aortic valve stenosis, myocardial infarction and myocarditis.^287^ MC depletion led to reduced cardiac function and suppressed cardiomyocyte contractility after MI.^288^ Among lymphocytes, CD4^+^ T cells indirectly enhance fibrosis by promoting macrophage polarization,^289^ which further activates cardiac fibroblasts and enhances wound healing after myocardial infarction.^290^ In a model of I/R injury, CD4^+^ depletion but not CD8^+^ depletion significantly reduced the infarct size.^291^ Regulatory T cells (Tregs) recruitment was observed in infarcted myocardium and Tregs ablation after MI leaded to enhanced inflammatory response and fibrosis. In vitro study showed that Tregs could directly modulate phenotype of cardiac fibroblast.^292^ In addition another study also proved that Tregs could modulate monocyte/macrophage differentiation.^293^ B cells are involved in the regulation of atherosclerosis, chronic heart failure, and cardiac remodeling after injury.^294^ Zouggari et al. observed mature B cell recruitment in the ischemic myocardium and B cell depletion had decreased inflammatory responses and improved cardiac function after MI. Moreover, impaired monocyte mobilization and compartmentalization were observed after B cell depletion.^295^ Natural killer (NK) cells have been suggested to prevent fibrosis and inflammatory cell maturation in the heart.^283^ Ong et al. depleted NK cells in mice via injection of anti-asialo GM1 antibody. These mice had increased inflammatory response and cardiac fibrosis in a model of autoimmune myocarditis.^296^ In this review, we mainly focus on the associated signaling pathways in immune cells.

#### Macrophages

##### Membrane receptors on macrophages

Tyro3, AXL and MerTK (collectively known as TAM receptors) make up a family of receptor tyrosine kinases and are expressed on the surface of macrophages, and these receptors are involved in efferocytosis and the inflammatory response.^297^ MerTK was shown to mediate the efferocytosis of apoptotic cardiomyocytes to promote inflammation resolution and cardiac repair after myocardial infarction.^298^ In addition, myocardial I/R initiates CCR2-dependent monocyte recruitment, which leads to MerTK cleavage and impairs MerTK function, resulting in impaired cardiac repair.^299^ In chronic heart failure patients, AXL was upregulated in the myocardium and the circulation.^300^ A recent study showed that AXL levels were elevated on cardiac macrophages in both humans and mice after I/R injury. In cardiac macrophages, enhanced crosstalk between AXL and Toll-like receptor 4 (TLR4) promoted STAT1 phosphorylation and the subsequent activation of HIF-1α signaling. This cascade led to a metabolic transition into glycolysis in cardiac macrophages and the secretion of IL-1β, which contributed to intramyocardial inflammation, adverse ventricular remodeling, and impaired contractile function.^301^ LGR4 is a member of the leucine-rich repeat-containing G protein-coupled receptor (LGR) family and is highly expressed on the membranes of cardiac macrophages after myocardial infarction. Specifically, LGR4 binds to Gαs, activates the cAMP/PKA/CREB cascade, and promotes CREB-mediated transactivation of the Fos gene family, subsequently resulting in enhanced activity of AP-1, which regulates the transcription of a series of inflammatory genes. This process eventually activates the proinflammatory activity of macrophages.^302^ A study published in 2020 further expanded the role of macrophages in maintaining physiological heart function. Cardiomyocytes eliminated dysfunctional mitochondria and other cellular garbage by packaging them in membranous particles known as cardiac exophers, which was dependent on cardiomyocyte autophagy. The released exophers were actively taken up and digested by cardiac macrophages via the phagocytic receptor MerTK. These processes were enhanced under stressed conditions, such as myocardial infarction. Depletion of cardiac macrophages or MerTK knockout led to cardiac dysfunction by triggering several pathological events: impaired elimination of mitochondria, the accumulation of dysfunctional mitochondria in cardiomyocytes, impaired autophagy, and inflammasome activation.^303^

Apart from macrophage-specific receptors, Dectin-1, a member of the C-type lectin receptor family that is mainly expressed on activated myeloid cells, was also shown to be a crucial regulator of macrophage function in I/R injury. The expression level of Dectin-1 in the myocardium was upregulated after myocardial I/R injury. Specifically, Dectin-1 deficiency or inhibition improved cardiac function after I/R injury and decreased M1 macrophage polarization and neutrophil infiltration. Dectin-1 contributes to neutrophil infiltration by regulating the expression of chemokine C-X-C motif ligand 1 (CXCL1) and granulocyte colony-stimulating factor (G-CSF). In addition, the production of IL-23/IL-1β in Dectin-1-induced M1 macrophages triggered the secretion of IL-17A by γδT cells, which was also involved in neutrophil infiltration. These signaling pathways eventually resulted in activation of the inflammatory response in heart injury.^304^ Other receptors, like angiotensin II receptor type 1 and adenosine 2 A receptor, mediated the role of macrophage in vascular injury.^305,306^

##### Intracellular signaling in macrophages

Phagocytosis activates Smad3 in the absence of TGF-β. The activation of Smad3 in turn accelerates milk-fat globule EGF factor-8 (MFGE8) synthesis, which initiates anti-inflammatory cascades, contributes to the anti-inflammatory phenotype of macrophages, and ultimately protects the heart from myocardial infarction. Smad3-mediated proinflammatory effects are also associated with changes in phagocytosis-induced peroxisome proliferator-activated receptor (PPAR) expression.^307^

##### Interaction between macrophages and other cardiac cells

M1-like macrophages were shown to worsen cardiac dysfunction after myocardial infarction. In the context of myocardial infarction, M1-like macrophages secrete proinflammatory exosomes, which transfer miR‑155 to endothelial cells. miR‑155 was taken up and led to angiogenesis inhibition and cardiac dysfunction by downregulating the target genes Rac family small GTPase 1 (RAC1), p21-activated kinase 2 (PAK2), Sirtuin 1, and AMPKα2.^308^ miR-155-containing exosomes can also be taken up by cardiac fibroblasts and inhibit the expression of SOS1 to inhibit fibroblast proliferation and suppressor of cytokine signaling 1 (SOCS1) expression to enhance the inflammatory response.^309^ Iron balance is crucial for maintaining normal cardiovascular conditions. Iron deficiency^310^ and overload^311^ both contribute to cardiovascular disease. Hepcidin, a sensor and regulator of iron, is synthesized and secreted by a subpopulation of inflammatory cardiac macrophages, and the specific loss of hepcidin in macrophages triggers cardiomyocyte renewal and cardiac regeneration after myocardial infarction in adult mice and apical resection in neonatal mice. Hepcidin suppressed the secretion of the reparative cytokines IL-4 and IL-13 via the phosphorylation of STAT3 in macrophages.^312^

IL-35 is mainly produced by regulatory Tregs. IL-35 was upregulated in myocardial infarction, which promoted cardiac healing. IL-35 neutralization led to impaired wound healing, increased cardiac rupture rate and impaired cardiac function. Mechanistically, IL-35 increased reparative macrophage survival to maintain cardiac recovery by activating STAT1 and STAT4 to enhance the expression of CX3C chemokine receptor 1 (CX3CR1) and TGF-β1.^313^

#### Neutrophils

The inflammatory response after heart transplantation contributes to graft failure. Ferroptosis was shown to be an important initiation factor in the posttransplantation inflammatory response. Ferroptosis recruits neutrophils to the injured myocardium by promoting neutrophil adhesion to coronary endothelial cells via the TLR4/TIR-domain-containing adapter-inducing interferon-β (TRIF)/type I interferon (INF) pathway. Inhibiting ferroptosis reduced cardiomyocyte death and neutrophil recruitment after transplantation.^314^ Neutrophil infiltration and the rate of early onset myocardial rupture were decreased by cardiomyocyte-specific TGF-β receptor knockout in myocardial infarction. TGF-β inhibition in cardiomyocytes augments the secretion of multiple protective cardiokines, including thrombospondin 4 (Thbs4), IL-33, follistatin-like 1 and growth and differentiation factor 15. These cardiokines play inhibitory roles in neutrophil integrin activation and tissue migration.^315^ Cardiac infiltration of neutrophils also plays a role in pressure overload-induced heart failure. Myeloid deficiency of Wnt5a, a noncanonical Wnt ligand, suppressed neutrophil infiltration in the heart and pathological remodeling after TAC surgery. The depletion of neutrophils produced a similar phenotype. In contrast, Wnt5a overexpression led to exacerbated cardiac hypertrophy, inflammation, and cardiac dysfunction.^316^

#### MCs

In myocardial infarction, MCs infiltrate the heart primarily through white adipose tissue. MC depletion led to impaired cardiac function after myocardial infarction mainly due to the suppression of cardiomyocyte contractility. MC-specific protease regulated PKA activity in cardiomyocytes via protease-activated receptor 2 proteolysis, which in turn regulated myofilament phosphorylation and Ca^2+^ sensitization.^288^

### Vascular endothelial cells

Vascular endothelial cells (VECs) are the most abundant noncardiomyocytes in the heart. In an adult mouse heart, it is estimated that 60% of noncardiomyocytes are VECs.^317^ Apart from forming the inner layer of the vascular network, VECs also play important roles in the regulation of vessel tone, inflammation, immune cell adhesion and migration, smooth muscle proliferation, and coagulation.^318^ Furthermore, VECs participate in maintaining cardiomyocyte homeostasis. In the heart, the myocardial capillary endothelium and coronary vascular endothelium have very distinct locations and, therefore, have different effects on the heart. Coronary VECs are in close contact with vascular smooth muscle cells and are mainly involved in regulating vascular tone. Coronary VECs could also affect cardiac function indirectly through coronary perfusion. In contrast, myocardial capillary VECs are in immediate contact with cardiomyocytes. The short distance and anatomical arrangement between capillary ECs and cardiomyocytes create an optimal condition for intercellular communication.^319,320^ Indeed, an in vitro study showed that when neonatal rat cardiomyocytes were cocultured with bovine aortic endothelial cells, there was a 2.1-fold increase in atrial natriuretic factor secretion.^321^ In vivo studies showed that VECs were important in maintaining normal myocardial contractile function.^322,323^ On the other hand, contact with cardiomyocytes is also necessary for the secretory function of VECs.^324^ These data suggest that dysfunction in VEC-cardiomyocyte communication will negatively affect myocardial physiology and predispose patients to maladaptive remodeling and even the progression of chronic heart failure. Unfortunately, pathological factors in chronic heart failure, such as ROS, exert harmful effects on VEC function.^325^ The most established molecules that transduce signals from VECs to cardiomyocytes are nitric oxide (NO) and endothelin (ET). There is evidence that VECs can secrete many other factors that are dysregulated during chronic heart failure and are involved in cardiac remodeling, such as IL-6, periostin, and tenascin-C,^326^ and release extracellular vesicles to transduce signals to neighboring cells in the heart,^327,328^ but there has been a relatively limited number of studies on this topic. Therefore, this review mainly focuses on NO and ET.

#### NO

In 1980, Furchgott and Zawandski identified a substance that was secreted by ECs that had a local vasodilation effect,^329^ which was initially named endothelium-derived relaxation factor (EDRF). Later, EDRF was identified as nitric NO.^330^ Although other mechanisms cannot be excluded, NO elicits effects on various cell types by activating soluble guanylyl cyclase (sGC), which increases cGMP levels. The accumulation of cGMP can in turn activate multiple downstream targets, such as protein kinases, ion channels, and cGMP-dependent PDEs.^331^ This signaling pathway has been shown to exert a protective effect against cardiac remodeling. An in vitro study showed that the NO donor S-nitroso-N-acetyl-D,L-penicillamine attenuated the norepinephrine-induced increase in protein synthesis in both cardiomyocytes and fibroblasts. The cGMP analog 8-bromo-cGMP has a similar suppressing effect.^332^ Multiple in vivo studies showed that the administration of NO sources inhibited cardiac remodeling after myocardial infarction or hypertension.^333–335^ Moreover, NO was shown to be an important mediator of the beneficial effects of angiotensin-converting-enzyme inhibitor treatment.^336^ Multiple molecular mechanisms have been proposed to explain the cardioprotective effect of NO and the cGMP pathway. For example, cGMP-activated protein kinase G (PKG) directly phosphorylates tuberin to block the activation of target of rapamycin complex-1 (mTORC1) under cardiac stress.^337^ PKG also phosphorylates ion channels and attenuates Ca^2+^ entry, thus inhibiting activation of the calcineurin/NFAT hypertrophic signaling pathway.^338^ We refer readers to another review for a more detailed description of the cGMP/PKG signaling pathway.^339^

Reduced bioavailability of NO is the hallmark of endothelial dysfunction in various cardiovascular diseases.^340^ Several approaches to enhance NO signaling in chronic heart failure have been tested. The earliest attempt was to increase NO by NO sources, such as inorganic nitrate. Before the establishment of standard neurohormonal blockade therapy, the combination of hydralazine and isosorbide dinitrate reduced mortality risk in male chronic heart failure patients.^341^ There is also evidence that the combination of hydralazine and isosorbide dinitrate still provides benefits in black patients, in addition to standard chronic heart failure treatment.^342^ However, tolerance and resistance are the major shortcomings. After continuous application, the effects of nitrates vanish.^343,344^ Therefore, current heart failure guidelines do not recommend routine use of nitrates in chronic heart failure patients.^345^ The second approach was to prevent cGMP breakdown. PDEs are a family of enzymes that catalyze the degradation of cAMP and cGMP into 5’-AMP and 5’-GMP, respectively. Among them, PDE5 specifically acts on cGMP. Sildenafil was developed to inhibit PDE5 and thereby enhance the NO-cGMP signaling pathway. This compound is now approved as a treatment for erectile dysfunction and pulmonary arterial hypertension.^346,347^ However, the treatment efficacy for chronic heart failure was disappointing. In the RELAX trial, sildenafil did not improve exercise capacity or clinical status among patients with heart failure with preserved ejection fraction (HFpEF).^348^ The neutral results could be explained by a following metabolites profiling study. This study indicated that sildenafil treatment might have adverse effect on mitochondrial function and endoplasmic reticulum stress through an unknown mechanism.^349^ The latest encouraging news comes from the third approach: sGC stimulation. The novel oral sGC stimulator vericiguat sensitizes sGC to nitric oxide by stabilizing nitric oxide binding to the binding site.^350^ In the VICTORIA trial, vericiguat improved the prognosis of patients with heart failure with reduced ejection fraction.^351^ The success of the VICTORIA trial builds confidence in the future exploration of novel medications targeting the NO/cGMP pathway.

#### ET

ET is a short peptide that was discovered in the culture media of bovine aortic ECs in 1985.^352,353^ Three ET peptides (ET-1, ET-2, and ET-3) were identified, and ET-1 is the major peptide in the cardiovascular system. Multiple factors in chronic heart failure could induce ET-1 gene expression in ECs, such as inflammatory cytokines, Ang II and TGF-β.^354^ Two G protein-coupled ET receptors have been identified (ET receptor type A (ETA) and ET receptor type B (ETB)). ETA is mainly expressed in the vasculature, heart, lungs, ovaries, and uterus and is believed to mediate most of the effects of ET-1.^355^ ET-1 is a strong vasoconstrictor.^352,353^ Together with NO and other vasoactive agents, ET-1 plays an important role in maintaining physiological vessel tone. When ET-1 binds to ETA on vascular smooth muscle cells, the coupled G protein activates PLC, which leads to the accumulation of IP3 and DAG. IP3 promotes calcium release from the endoplasmic reticulum and thus vasoconstriction.^356,357^ DAG stimulates the protein PKC. Activated PKC stimulates the Na+–H+ exchanger, which leads to a decrease in intracellular pH and the sensitization of contractile proteins to Ca^2+^.^358^ The same signaling pathway could also explain its inotropic effect on the heart.^358^ ET-1 also has features of growth factors. In the 1990s, exogenous ET-1 was suggested to have a hypertrophic effect on cultured cardiomyocytes.^359–361^ Cardiomyocyte-specific overexpression postnatally of ET-1 induced severe cardiac hypertrophy and the rapid deterioration of cardiac function.^362^ The hypertrophic effect of ET-1 is mostly mediated by increases in DAG. DAG, probably through PKC activation, activates the small G-protein Ras and, in turn, the downstream MAPK signaling pathway.^363,364^ In addition, ET-1 may also contribute to the progression of chronic heart failure by promoting cardiac fibrosis.^365–368^

Previous work led to the development of ETA antagonists for their therapeutic potential. BQ-123 is a peptide ETA antagonist that was discovered in 1992.^369^ Using BQ-123 for ETA blockade yielded promising results. In a rat model of myocardial infarction, although acute administration of BQ-123 decreased myocardial contractility, long-term ETA blockade improved rat survival and reduced myocardial remodeling.^370^ In a heart failure model induced by pressure overload, BQ-123 attenuated cardiac hypertrophy during the early phase.^371^ Orally active, small-molecule ET receptor antagonists have also been discovered. These agents included the earliest mixed ETA/ETB antagonist bosentan and others with differential ETA/ETB specificity.^372^ Short-term administration of bosentan did show beneficial hemodynamic changes in chronic heart failure patients.^373^ However, the following clinical trials were disappointing. The ENABLE trial showed that treatment with bosentan did not reduce the risk of the primary outcome in chronic heart failure patients. In contrast, bosentan appeared to increase the early risk of heart failure hospitalization because of fluid retention.^374^ Human clinical trials with enrasentan and ETA-selective darusentan were also unsuccessful.^375,376^ Interestingly, a follow-up mechanistic study showed that sympathetic neuron-specific but not cardiomyocyte-specific ETA knockout reduced cardiac remodeling induced by pressure overload. ETA inhibition in sympathetic neurons could attenuate adrenergic neurotransmission and exert cardioprotection.^377^ The authors proposed that the widely used beta-blocker might interfere with the beneficial effect of ET receptor antagonists in clinical trials with chronic heart failure patients.^377^

### Lymphatic endothelial cells (LECs)

The lymphatic vasculature maintains interstitial fluid balance^378^ and plays an important role in the immune response by transporting pathogens, antigens, and immune cells. The lymphatic vasculature contributes to the physiological function of multiple organs, including the heart. Recent studies have shown that the cardiac lymphatic vasculature undergoes massive remodeling under pathological conditions, suggesting that the lymphatic system is also involved in the cardiac response to injury. After myocardial infarction, extensive lymphangiogenesis was observed in the infarcted region.^379–381^ Vascular endothelial growth factor-C (VEGFC) can promote lymphatic vessel growth by interacting with VEGF receptor 3 (VEGFR3) but can also negatively affect blood vessels by interacting with VEGFR2.^382,383^ VEGFC-C156S is a point mutant of VEGFC that can selectively interact with VEGFR3.^384^ VEGFC-C156S can effectively induce the growth of lymphatic vasculature without affecting blood vessels.^383,385,386^ Augmentation of lymphangiogenesis during cardiac injury using VEGFC-C156S can reduce cardiac remodeling and improve heart function. Intraperitoneal injection of recombinant VEGFC-C156S, intramyocardial injection of microparticles loaded with VEGFC-C156S, and intraperitoneal injection of adeno-associated virus (AAV) encoding VEGFC-C156S all showed robust protective effects after myocardial infarction. These therapies improved cardiac function and reduced cardiac fibrosis and infarct size after MI.^379,380,387,388^ Researchers attributed the beneficial effect of lymphangiogenesis to improved fluid balance and enhanced immune cell clearance.^379,387–390^ Interestingly, a recent study further expanded our understanding of the function of cardiac lymphatic vessels.^381^ In this study, lymphatic endothelial cell-specific deletion of PROX1 (PROX1^ΔLEC/Δ LEC^) disrupted the development of the lymphatic vasculature. Heart weight was significantly reduced in PROX1^ΔLEC/Δ LEC^ mice due to reduced cardiomyocyte proliferation. This effect was independent of hemodynamic defects but was mediated by Reelin, a factor secreted by LECs. Reelin binds to integrin-β1 on cardiomyocytes and stimulates downstream signals such as FAK, DAB1, AKT and ERK. In an MI model in neonatal mice, Reelin deficiency led to reduced recovery due to suppressed cardiomyocyte proliferation and increased apoptosis. In adult hearts, local Reelin delivery by collagen patches reduced cardiac remodeling and improved cardiac function. In the latter case, Reelin reduced cardiomyocyte apoptosis but did not affect adult cardiomyocyte proliferation. This study demonstrated that in addition to its conventional role in fluid balance and the immune response, the lymphatic vasculature can also modulate the cardiac response to injury by paracrine signaling.

### HFpEF

Most of the abovementioned studies focused on HFrEF. During the past 2 decades, HFpEF has received increasing attention, along with the increase in the prevalence of diabetes and obesity. Although lacking a uniform definition,^391^ HFpEF generally refers to a group of patients with the classic symptoms of heart failure, a left ventricular ejection fraction in the normal range, and without a well-defined etiology, such as cardiac amyloidosis. HFpEF makes up 50% of all chronic heart failure cases,^392^ with a similar or slightly better prognosis than HFrEF.^392^ However, in the same context of chronic heart failure, HFpEF is distinct from HFrEF, not only in their demographic features and comorbidity patterns^393^ but also in their responses to pharmacological treatment. Standard neurohormonal blockade treatment did not improve the prognosis of HFpEF patients in large clinical trials.^394–398^ In 2021, the publication of the EMPEROR-Preserved trial announced the first piece of good news.^399^ The sodium–glucose cotransporter 2 inhibitor (SGLT2i) empagliflozin reduced the risk of the primary outcome by 21% in HFpEF patients, which was mainly driven by the reduced risk of hospitalization for heart failure. However, SGLT2i was originally designed to treat diabetes. The application of SGLT2i in chronic heart failure was derived from the unexpected reduction in heart failure risk in diabetes patients treated with SGLT2i.^18^ Therefore, to develop targeted therapy for HFpEF in the future, the pathophysiology and molecular mechanism need to be better understood, which turns out to be rather difficult.

Previously, HFpEF was defined as diastolic heart failure because diastolic dysfunction was believed to be the major reason for heart failure symptoms.^400^ However, it was gradually recognized that diastolic dysfunction was not a specific feature of HFpEF.^401–403^ Instead, the pathophysiology of HFpEF is much more complicated, including arterial stiffening and adverse ventricular–vascular interactions,^404,405^ chronotropic incompetence,^406–408^ pulmonary hypertension and right HF.^409,410^ In addition to the heart, HFpEF patients often have dysfunction in multiple organs, such as the kidney, lung, and skeletal muscle.^411–413^ These characteristics make HFpEF highly heterogeneous^414,415^ and have multiple subphenotypes.^416–418^ This complexity makes HFpEF hard to model in animal experiments.^419^ Currently available animal models only recapitulate some but not all features of HFpEF patients. As hypertension is highly prevalent in HFpEF, hypertension-induced left ventricle remodeling is traditionally believed to be the fundamental step in HFpEF pathophysiology.^420^ As a result, early HFpEF models emphasized hypertension. These models included Dahl salt-sensitive rats fed by a high-salt diet,^421^ spontaneously hypertensive rats,^422^ and mice with aortic constriction^422^ or Ang II infusion.^423^ These models had preserved ejection fraction and impaired diastolic function, but they eventually progressed to a decompensated stage where systolic function was also compromised.^419^ In HFpEF patients, however, LVEF is mostly stably preserved for a long time.^424^ Obesity and diabetes were also prevalent comorbidities in HFpEF. Multiple attempts were made to incorporate these 2 diseases into HFpEF models. Examples included obese Zucker diabetic fatty/spontaneously hypertensive rat hybrids.^425^ However, these animals benefited from β-blocker treatment,^426^ which is not the case in humans. A more recent pathogenic model of HFpEF proposed that comorbidities induced a systematic proinflammatory state, which caused coronary endothelial inflammation and dysfunction. NO-cGMP-PKG signaling was thus reduced in cardiomyocytes, promoting pathological cardiomyocyte hypertrophy and stiffness.^411^ The most recent two-hit HFpEF mouse model used a combination of a high-fat diet and constitutive nitric oxide synthase inhibition using Nω-nitro-L-arginine methyl ester (L-NAME).^427^ A high-fat diet induced metabolic stress, while L-NAME induced NO deficiency-based hypertension. This model was comprehensively characterized with multiple assays and recapitulated many features of HFpEF in humans. Using this model, researchers found that inducible nitric oxide synthase (iNOS) was increased in HFpEF hearts. Nitrosative stress causes S-nitrosylation of the endonuclease inositol-requiring protein 1α (IRE1α) and decreases its splicing activity on X-box-binding protein 1 (XBP1s), leading to a decrease in the spliced form of XBP1, which may affect stress responsiveness in cardiomyocytes.^428^ Pharmacological or genetic suppression of iNOS rescued cardiac dysfunction in this two-hit HFpEF model. However, the increase in iNOS was likely induced by the use of constitutive nitric oxide synthase inhibitors. It is not clear whether nitrosative stress plays an equally important role in HFpEF patients. Moreover, studies have shown that HFpEF is heterogeneous and has multiple subphenotypes with differences in clinical characteristics, cardiac function and structure, prognosis, transcriptome, and even response to treatment.^416,417,429,430^ It is unknown whether one mouse model can represent all these subphenotypes. The two-hit model was originally developed using male mice. However, researchers later found that female mice developed a milder cardiac phenotype than males, and the protective effect persisted even after ovariectomy.^431^ In an epidemiological study, however, elderly postmenopausal women had an equal, if not higher, risk of HFpEF than men.^432,433^ This inconsistency again emphasizes that a single animal model may not fit all HFpEF patients.

A recent transcriptome study of endomyocardial biopsy samples from HFpEF patients shed light on the molecular signaling in this disease.^429^ Compared with those in the control, inflammatory and immune response and oxidative phosphorylation pathways were upregulated in HFpEF hearts, while epigenetic modulators, membrane morphogenesis/organization, organonitrogen signaling, and receptor-coupled kinase signaling pathways were downregulated. Compared with HFrEF, HFpEF has higher expression of genes in the oxidative phosphorylation pathway but lower expression of genes in the endoplasmic reticular, cGMP-related, autophagy, fibrosis, and hypertrophy-related pathways. Using Non–negative matrix factorization, the author examined the subphenotypes of HFpEF based on transcriptome data. Three groups were identified. Group 1 had a higher proportion of males and diabetes with a lower BMI and LVEF and higher NT-proBNP levels and left ventricle dimensions. The transcriptomes of Group 1 were close to those of HFrEF. Group 2 included females with the highest BMI, smallest left ventricle dimensions and lowest NT-proBNP levels. The transcriptome of Group 2 was characterized by an enhanced immune-related pathway. Group 3 had higher expression of inflammatory and extracellular matrix process-related genes. This study highlighted the heterogeneity of molecular signaling in different HFpEF phenotypes.

## Emerging therapeutic strategies for failing heart

**Fig. 4:**
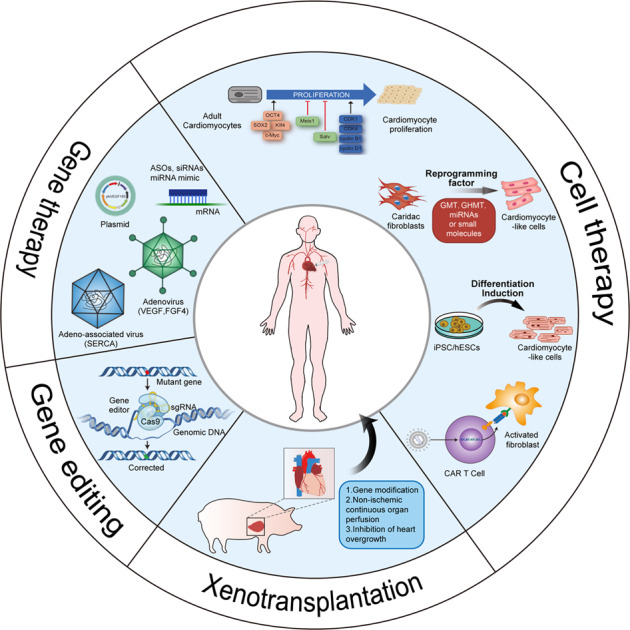
Emerging therapeutic strategies to treat failing hearts. Gene therapy via various vectors enables direct manipulation of gene expression. Cell therapy is aimed at replacing the lost functional cardiomyocytes either exogenously or endogenously. Chimeric antigen receptor (CAR) T cell therapy is also proposed to ameliorate cardiac fibrosis and improve cardiac function. Xenotransplantation technique makes pig-to-human heart transplantation possible by overcoming multiple cross-species barriers. Clustered regularly interspaced short palindromic repeats CRISPR)/CRISPR-associated 9 (Cas9) based gene editing has the potential for curing inherited cardiomyopathy in the future. CDK: cyclin-dependent kinase; GTM(H): Gata4, Mef2c, and Tbx5 (Hand2); iPSC: induced pluripotent stem cell; hESC: human embryonic stem cell; CAR T-cell: Chimeric Antigen Receptor T-Cell; sgRNA: small guide RNA; VEGF: vascular endothelial growth factor; FGF4: fibroblast growth factor 4; ASO: antisense oligonucleotide; SERCA: sarco/endoplasmic reticulum Ca2+−ATPase

Modern biomedical techniques have changed the landscape of future chronic heart failure treatment (Fig. 4). Pharmaceutical targeting of a disease-regulating protein is no longer the sole option. Virus-based and modified RNA-based in vivo delivery techniques enable the development of gene therapy. Progress in stem cell manipulation has given rise to cell therapy. The discovery of genetic editing techniques boosts research on heart xenotransplantation and the genetic correction of hereditary disease. We will summarize these breakthroughs in cardiomyopathy therapies. Representative clinical trials involved in this section was summarized in Table 1.

**Table 1:** Representative clinical trials of different therapeutic strategies

Study (PMID in NCBI)	Publication year	Phase	Sample size	Disease	Intervention	Endpoint	Main conclusion
**I. Gene therapy**							
**1. Plasmid-based**							
Losordo et al. (9860779)	1998	I	5	Angina	Myocardial injection of phVEGF plasmid	Safety	Myocardial injection of phVEGF is safe.
Sarkar et al. (11887971)	2001	I	7	Angina	Myocardial injection of phVEGF plasmid	Safety	Myocardial injection of phVEGF is safe.
Euroinject One trial (15808751)	2005	II	40/40	Ischemic heart disease	Myocardial injection of phVEGF plasmid or placebo	Myocardial stress perfusion defect assessed by SPECT	Myocardial injection of phVEGF plasmid did not improve stress-induced myocardial perfusion abnormalities.
NORTHERN trial (19352324)	2009	II	48/45	Coronary artery disease	Myocardial injection of phVEGF plasmid or placebo	Myocardial perfusion assessed by SPECT	Myocardial injection of phVEGF plasmid did not improve myocardial perfusion.
KAT (12742981)	2003	II	37/28/38	Coronary heart disease	Intracoronary infusion of VEGF adenovirus, phVEGF plasmid liposome or Ringer’s solution	Minimal lumen diameter and percent diameter stenosis measured by quantitative coronary angiography	The VEGF gene transfer did not significantly change restenosis rate or minimal lumen diameter.
Penn et al. (23429605)	2013	I	17	Ischemic cardiomyopathy	Endomyocardial injection of SDF-1 plasmid	Safety	Endomyocardial injection of pSDF-1 was safe.
STOP-HF trial (26056125)	2015	II	32/30/31	Ischemic heart failure	Endomyocardial injections of 15 or 30 mg dose of SDF-1 plasmid or placebo	Change of a composite endpoint consisting of the 6 MWD and MLWHFQ	Endomyocardial injection of SDF-1 plasmid did not improve change in 6 MWD and MLWHFQ
**2. Virus-based**							
Rosengart et al. (10430759)	1999	I	21	Coronary artery disease	Myocardial injection of Ad-VEGF	Safety	Myocardial injection of Ad-VEGF was safe.
REVASC trial (16791287)	2006	II	32/35	Angina	Myocardial injection of Ad-VEGF or continuation of medical treatment	Exercise time to 1 mm ST-segment depression	Myocardial injection of Ad-VEGF increased exercise time to 1 mm ST-segment depression.
NOVA trial (21252014)	2011	II	12/5	Coronary artery disease	Myocardial injection of Ad-VEGF or placebo	Change in total exercise duration	The study was prematurely terminated. Myocardial injection of Ad-VEGF did not improve exercise capacity.
AGENT (11901038)	2002	II	60/19	Angina	Intracoronary infusion of Ad5-hFGF or placebo	Exercise treadmill testing	Intracoronary infusion of Ad5-hFGF could exercise time.
AGENT 2 (14563572)	2003	II	35/17	Angina	Intracoronary infusion of Ad5-hFGF or placebo	Reversible perfusion defect size assessed by SPECT	Intracoronary infusion of Ad5-hFGF improved myocardial perfusion.
AGENT 3 (17825712)	2007	IIb/III	140/137/139	Angina	Intracoronary infusion of high-dose, low-dose of Ad5-hFGF or placebo	Exercise tolerance test	The study was prematurely terminated. Intracoronary infusion of Ad5-hFGF did not improve exercise capacity.
AGENT 4 (17825712)	2007	IIb/III	35/43/38	Angina	Intracoronary infusion of high-dose, low-dose of Ad5-hFGF or placebo	Exercise tolerance test	The study was prematurely terminated. Intracoronary infusion of Ad5-hFGF did not improve exercise capacity.
Hammond et al. (27437887)	2016	II	42/14	Heart failure	Intracoronary infusion of Ad5-hAC6 or placebo	LVEF, LV peak +dP/dt and peak –dP/dt before and during dobutamine infusion, and exercise capacity	Intracoronary infusion of Ad5-hAC6 improved LV function.
CUPID (21709064)	2011	II	9/8/8/14	Heart failure	Intracoronary infusion of high-dose, mid-dose, low-dose of AAV1/SERCA2a, or placebo	Composite endpoints of measurements in symptomatic, functional, biomarker, LV function/remodeling domains	Intracoronary infusion of AAV1/SERCA2a could improve advanced heart failure.
CUPID 2 (26803443)	2016	IIb	123/127	Heart failure	Intracoronary infusion of AAV1/SERCA2a or placebo	Time to hospital admission because of heart failure or ambulatory treatment for worsening heart failure	Intracoronary infusion of AAV1/SERCA2a did not improve prognosis.
AGENT-HF (28393439)	2017	II	5/4	Heart failure	Intracoronary infusion of AAV1/SERCA2a or placebo	Change in LVESV	The study was prematurely terminated. Intracoronary infusion of AAV1/SERCA2a did not improve LV remodeling
SERCA-LVAD (32669717)	2020	IIa	4/1	Heart failure with a LVAD	Intracoronary infusion of AAV1/SERCA2a or placebo	Exercise capacity	The study was prematurely terminated.
**3. Oligonucleotide-based**							
Fitzgerald et al. (27959715)	2017	I	24	Healthy volunteers	Subcutaneous injection anti-PCSK9 siRNA loaded in nanoparticles (inclisiran) or placebo	Safety	Subcutaneous injection of inclisiran was safe.
Ray et al. (28306389)	2017	II	Totally 501	Patients with elevated LDL-c	Subcutaneous injection of inclisiran or placebo	Change of LDL-c	Subcutaneous injection of inclisiran lowered LDL-c
ORION-10 trial (32187462)	2020	III	781/780	Atherosclerotic cardiovascular disease	Subcutaneous injection of inclisiran or placebo	Change of LDL-c	Subcutaneous injection of inclisiran lowered LDL-c by ~50%
ORION-11 trial (32187462)	2020	III	810/807	Atherosclerotic cardiovascular disease or an atherosclerotic cardiovascular disease risk equivalent	Subcutaneous injection of inclisiran or placebo	Change of LDL-c	Subcutaneous injection of inclisiran lowered LDL-c by ~50%
**II. Gene editing**							
Gillmore et al. (34215024)	2021	I	6	ATTR amyloidosis with polyneuropathy	Intravenous infusion of anti-TTR CRISPR-Cas9 system loaded in lipid nanoparticle (NTLA-2001).	Safety and change in serum TTR protein	Intravenous infusion of NTLA-2001 was safe and led to decrease in serum TTR protein
**III. Cell therapy**							
**1. Myoblast**							
Menasché et al. (12679204)	2003	I	10	Ischemic cardiomyopathy	Autologous skeletal myoblast transplantation	Safety	Autologous skeletal myoblast transplantation was safe, except for its arrhythmogenic potential.
MAGIC trial (18285565)	2008	II	67/30	Ischemic cardiomyopathy	Autologous skeletal myoblast or placebo transplantation	Global and regional LV function assessed by echocardiography	Myoblast injections combined failed to improve echocardiographic heart function.
**2. Bone marrow-derived mesenchymal stem cell**							
Yao et al. (18381377)	2008	II	24/23	Ischemic heart disease with previous myocardial infarction	Intracoronary infusion of bone marrow mononuclear cells	LV systolic and diastolic function, infarct size and myocardial perfusion	Intracoronary transfer of autologous bone marrow mononuclear cells did not improve cardiac systolic function, infarct size or myocardial perfusion, but improved diastolic function.
MSC-HF trial (25926562)	2015	II	40/20	Ischemic cardiomyopathy	Intra-myocardial injections of bone marrow-derived mesenchymal stromal cell or placebo	LVESV and LVEF	Intra-myocardial injections of bone marrow-derived mesenchymal stromal cell improved myocardial function.
REPAIR-AMI (17620510)	2006	III	103/101	Acute myocardial infarction	Intracoronary infusion of bone marrow derived progenitor cells or placebo	Change in LVEF	Intracoronary infusion of bone marrow derived progenitor cells improved LV contractile function.

*hVEGF* human vascular endothelial growth factor, *SDF-1* stromal cell-derived factor 1, *6 MWD* 6-min walk distance, *MLWHFQ* Minnesota Living with Heart Failure Questionare, *Ad* adenovirus, *Ad5* adenovirus serotype 5, *hFGF* human fibroblast growth factor, *SPECT* single-ohoton emission computed tomography, *hAC6* human adenylyl cyclase 6, *AAV1* adeno-associated virus serotype 1, *SERCA2a* sarco/endoplasmic reticulum Ca2+-ATPase 2a, *LVAD* left ventricular assist device, *LDL-c* low-density lipoprotein cholesterol, *ATTR* transthyretin amyloidosis, *LVESV* left ventricular end-systolic volume, *LVEF* left ventricular ejection fraction.

### Therapeutic strategies based on gene therapy

**Fig. 5:**
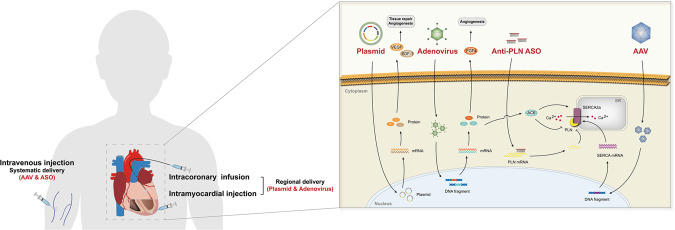
Summary of therapeutic strategies based on gene therapy for failing hearts. Early attempts were plasmid and adenovirus based. These reagents are usually locally delivered by intracoronary infusion or intramyocardial injection. The overexpressed genes encode secretory protein related to angiogenesis and tissue repair. In one trial, the putative protective adenylyl cyclase type 6 (AC6) was overexpressed. Recent gene therapy trials adopt adeno-associated virus (AAV). A series of clinical trials are designed to overexpress sarco/endoplasmic reticulum Ca^2+^-ATPase 2a (SERCA2a) in cardiomyocytes to improve Ca^2+^ handling in heart failure. Modified RNAs represent a novel approach to target specific genes. It’s normally systemically delivered. Although there hasn’t been any clinical trial testing RNA therapy for failing heart, preclinical studies using antisense oligonucleotide (ASO) against phospholamban (PLN) showed promising results. AAV: Adeno-associated virus; ASO: antisense oligonucleotide; FGF4: fibroblast growth factor 4; VEGF: vascular endothelial growth factor; SDF-1: stromal cell derived factor-1; SERCA: sarco/endoplasmic reticulum Ca2+-ATPase; PLN: phospholamban; AC6: Adenylyl cyclase type 6

Gene therapy aims to modulate the expression of genes to treat diseases. The real understanding of gene therapy began in the 1960s ~1980s, when the genetic code was deciphered,^434,435^ and AAV^436^ and a series of enzymatic tools were identified.^437,438^ The first clinical gene therapy trial (ADA-SCID) was conducted in 1990 and was aimed at treating patients with adenosine deaminase deficiency.^439^ During the past 30 years, nearly three thousand clinical trials on gene therapy have been conducted, and seven products have been approved by the FDA or European Medicines Agency according to the Gene Therapy Clinical Trials Worldwide database.^440^ In the field of cardiac disease, although most gene therapy trials yielded negative results, they provided valuable evidence for future studies (Fig. 5).^441^

#### Plasmid-based gene therapy

Plasmids can package large DNA fragments and are easy to manipulate and store. Importantly, plasmids have low tumorigenicity due to rare genomic integration.^442^ In 1996, the first plasmid-mediated gene therapy was conducted in rabbits. phVEGF165 (cDNA encoding the 165-amino acid isoform of VEGF inserted into the pUC118 vector) was injected into the ischemic hindlimb muscles of rabbits and improved collateral circulation and tissue perfusion.^443^ The clinical application of phVEGF165 was soon evaluated in patients with ischemic limbs. In total, 2000 µg of phVEGF165 was infused into the popliteal artery by an angioplasty balloon, which promoted angiogenesis.^444^ Initial plasmid-mediated gene therapy in peripheral arterial disease yielded promising outcomes in clinical studies.^445^ In 1998, phVEGF165 gene therapy was used to promote myocardial angiogenesis in patients with symptomatic myocardial ischemia. In this study, a low dose of 125 μg phVEGF165 was directly injected into the ischemic myocardium through a mini left anterior thoracotomy. Myocardial perfusion was also improved.^446^ A similar study using a higher dose of 250 μg showed similar results.^447^ However, these trials were open-label, single-arm trials without placebo controls. The observed beneficial effects could be biased by the limitation in the study design.^440^ Indeed, subsequent studies with double-blinded placebo-controlled designs failed to show positive results.^448–450^ Stromal cell-derived factor-1 (SDF-1) is a regulator of tissue repair that has multiple beneficial effects on ischemic cardiomyopathy.^451,452^ The phase 2 STOP-HF trial was designed to determine the therapeutic potential of a plasmid encoding stromal cell-derived factor-1 (pSDF-1) in chronic ischemic heart failure. Ninety-three patients were randomized to receive placebo, 15 mg of pSDF-1, or 30 mg of pSDF-1. The primary endpoint of a composite change in the Minnesota Living with Heart Failure Questionnaire and 6-min walk distance was not met, but there were trends toward improvements in ejection fraction and left ventricular end-systolic volume.^453^ However, there hasn’t been any information about a subsequent phase 3 trial.

#### Virus-based gene therapy

Compared to the low delivery efficiency of plasmid vectors, adenoviruses and AAV vectors are more effective and efficient forms of gene delivery. The viral vectors consist of a protein capsid or a lipid envelope and carry the target cDNA inside, which can be delivered into the nucleus of the target cell.^454^

##### Adenoviral vectors

Adenovirus (Ad) is a double-stranded (ds) DNA viruses. With a 36 kb genome, it has a large packaging capacity. Ad is widely used for in vitro transfection of dividing and nondividing cells, including cardiomyocytes.^455^ Due to robust but transient gene expression, Ad vectors have been chosen as delivery tools for short-term revascularization in myocardial ischemia.^456^ In 1999, the first Ad-based phase I clinical trial was conducted to use gene therapy to promote cardiac angiogenesis. In this study, human VEGF 121 cDNA carried by adenovirus was administered by myocardial injection. In this trial, VEGF gene therapy successfully induced myocardial angiogenesis and improved exercise capacity in patients with coronary artery disease.^457^ However, later randomized controlled trials with larger sample sizes did not yield positive results.^458,459^ In addition to the transient nature of Ad-based overexpression, another shortcoming was the proinflammatory effect of Ad capsule proteins,^460^ which may lead to safety issues in clinical practice. Moreover, transfection of the Ad vector into nontarget cells was also a potential problem in cardiac gene therapy.^461^ To solve the problem of specificity, an adenovirus serotype 5 (Ad5)-derived recombinant vector was used as gene therapy for cardiac disease. Ad5 can recognize Coxsackie-adenovirus receptor (CAR) highly expressed on the surface of cardiomyocytes.^462^ Ad5 vectors were used to deliver fibroblast growth factor 4 (FGF4) in a series of angiogenic gene therapy (AGENT) clinical trials (from AGENT to AGENT4). Again, although AGENT and AGENT 2 showed some promising preliminary data,^463^ phase 2/3 AGENT 3 and 4, which had larger sample sizes, did not show any improvements in patients with stable angina, although subgroup analysis did show that certain patient subgroup might benefit from the therapy.^464^ Adenylyl cyclase type 6 (AC6) is the dominant AC in cardiomyocytes. Overexpression of AC6 preserved heart function in a genetic model of cardiomyopathy.^465,466^ The beneficial effect of AC6 is likely to be mediated by a cAMP-independent mechanism, such as improving Ca^2+^ mishandling.^467,468^ Therefore, a phase 2 study was designed to test the safety and efficacy of single intracoronary administration of Ad5 encoding human AC6 (Ad5.hAC6). Ad5.hAC6 therapy was shown to be safe. The treatment group with the highest viral dose had improved ejection fraction, LV peak -dP/dt, and heart failure symptoms compared with the placebo-control group.^469^ A larger phase 3 trial was designed and launched to further validate the efficacy of Ad5.hAC6 treatment in chronic heart failure.^470^

##### AAV vectors

In contrast to Ads, adeno-associated viruses (AAVs) are single-stranded DNA viruses and have been suggested to be more useful and suitable for in vivo gene delivery due to their safety, long duration of gene expression and mild immune response.^456^ AAV-based gene therapy has made exciting progress in multiple diseases, including retinal degenerative disorder and central nervous system diseases. Some therapies have been approved for clinical applications.^471^ An important advantage of the AAV vector is its tissue tropism.^472^ In chronic heart failure, Ca^2+^ handling in cardiomyocytes is important for contractility regulation.^440^ SERCA is a crucial regulator of cardiac Ca^2+^ cycling and contractility. SERCA2a, a spliced transcript of SERCA, is only expressed in cardiac muscle and slow twitch skeletal muscle.^473^ A preclinical study showed that SERCA2a gene delivery to cardiomyocytes improved heart function in models of heart failure.^474–476^ These promising results drove translation efforts in clinical trials. First, the phase 2 CUPID 1 trial tested the effects of AAV1-mediated SERCA2a on patients with advanced chronic heart failure. Thirty-nine patients were randomized to receive intracoronary infusion of placebo or different doses of AAV1-SERCA2a. AAV1-SERCA2a therapy was safe and showed a trend of beneficial effects.^477,478^ However, the subsequent phase 2b CUPID 2 trial yielded disappointing results. In total, 250 patients were randomized to receive AAV1-SERCA2a or placebo by intracoronary infusion. SERCA2a gene therapy did not reduce the risk of hospitalization for heart failure or ambulatory treatment for worsening heart failure.^479^ The AGENT-HF trial was a separate study from the CUPID II trial, which was prematurely terminated because of the neutral results of CUPID II. Analyses of the 9 patients involved did not indicate a beneficial effect of AAV1-SERCA2a in terms of the change in left ventricular end-systolic volume in chronic heart failure patients.^480^ SERCA-LVAD tested this therapy in chronic heart failure patients implanted with a left ventricular assist device. SERCA-LVAD was terminated after the publication of CUPID II and did not show beneficial effects.^481^ The level of delivered genes in myocardial tissues obtained from cardiac transplantation, biopsy, or autopsy was low or even undetectable in the CUPID, CUPID II, and SERCA-LVAD trials, which could be caused by either low delivery efficiency or lack of persistence. Pre-existing neutralizing antibodies could be one of the reasons. It’s estimated that 59.5% of heart failure patients had pre-existing AAV1 neutralizing antibodies in their serum.^482^ Prolonged expression of target genes appears to be a major goal in improving cardiac gene therapy.

#### Oligonucleotide-based gene therapy

The abovementioned gene therapy treats diseases based on the protective role of gene overexpression. Some diseases can also benefit from the downregulation of specific genes by targeting mRNAs. Although gene can be silenced by AAV-delivered short hairpin RNAs,^483^ this method is still limited to basic research. Alternatively, gene silencing can also be achieved by systematic administration of ASOs or siRNAs. Proper chemical modifications greatly improve the stability, distribution, cellular uptake, and potency of these agents.^484^ Compared with traditional pharmaceutical treatment, RNA therapy has some important advantages. First, some proteins are difficult to target with inhibitory molecules due to their specific conformation, while mRNAs can be easily targeted by oligonucleotides. Moreover, noncoding RNAs, which are traditionally considered undruggable, can also be targeted by RNA therapy. Second, off-target effects can be avoided by proper sequence design. Third, once the platform is established, a new oligonucleotide drug can be rapidly developed compared with the start from scratch approach of traditional drug development. RNA therapy has been successful in multiple diseases.^485^ The most typical example in the cardiovascular field is inclisiran for treating hypercholesterolemia. Proprotein convertase subtilisin–kexin type 9 (PCSK9) is a well-established target of LDL-C-lowering therapy. PCSK9 is mainly expressed in hepatocytes and secreted into the circulation. PCSK9 binds to and mediates LDL receptor degradation in hepatocytes, consequently reducing LDL-C uptake and increasing circulating LDL-C levels. Targeting PCSK9 with blocking antibodies in addition to standard therapy lowers LDL-C levels and reduces cardiovascular risk.^486,487^ Inclisiran is a synthetic siRNA against PCSK9 mRNA. Inclisiran is conjugated to triantennary N-acetylgalactosamine carbohydrates, which increases hepatocyte specificity via asialoglycoprotein receptors. The siRNA is modified with phosphorothioate, 2′-O-methyl nucleotide, and 2′-fluoro nucleotide modifications to improve molecular stability. In patients with elevated LDL-C despite maximum tolerated statin therapy, subcutaneous injection of inclisiran every 6 months reduced LDL-C levels by approximately 50%.^488–490^ The long-lasting LDL-C-lowering effect of inclisiran is a great advantage for increasing patient compliance. These data demonstrate the feasibility and efficacy of oligonucleotides in treating cardiovascular disease. Antisense technology has not been tested to treat cardiomyopathy in clinical trials, but preclinical studies have shown promising results. For example, in mouse models of dilated cardiomyopathy, subcutaneous injection of an ASO against PLN rescued Ca^2+^ mishandling and improved cardiac function.^491^ In addition to targeting protein-coding genes, targeting noncoding RNAs is also feasible. Wisp2 superenhancer–associated RNA (Wisper) is a cardiac fibroblast-enriched lncRNA that positively regulates cardiac fibrosis after injury. ASO-mediated downregulation of Wisper attenuated cardiac fibrosis after myocardial infarction and improved cardiac function.^492^ miRNA mimics are an alternative method for gene silencing. Compared with siRNAs or ASOs, miRNA mimics is are less specific and have multiple targets. However, this can be an advantage, as numerous genes are dysregulated in the failing heart.^493^ Intracardiac injection of miR-19a/19b mimics induced cardiomyocyte proliferation and cardiac regeneration after myocardial infarction in mice.^494^ However, there has not been a modification that enhances cardiomyocyte uptake of oligonucleotides. Increasing tissue specificity and reducing unwanted side effects will be a major obstacle in improving RNA therapy for cardiomyopathy.

### Therapeutic strategies based on gene editing

Inherited cardiomyopathies are functional and structural disorders of the heart caused by genetic defects, including but not limited to hypertrophic, dilated, restrictive, arrhythmogenic right ventricular cardiomyopathies. These diseases are often progressive and significantly reduce life expectancy. However, current treatments are often symptom relieving or palliative.^495–497^ With recent progress in gene-editing technology, correcting pathogenic gene mutations may be achieved in the near future. Although gene editing can be achieved by some technologies, such as zinc finger nucleases (ZFNs)^498^ and transcription activator-like effector nucleases (TALENs),^499^ the recent discovery of a clustered regularly interspaced short palindromic repeats (CRISPR)/CRISPR-associated 9 (Cas9) system boosted research in this field.^500^ CRISPR/Cas9 is derived from the CRISPR/Cas system in bacteria, which provides bacteria with adaptive immunity to viruses and plasmids. When bacteria are infected, a short sequence of invading DNA is inserted as a spacer sequence into the CRISPR array. The transcribed precursor CRISPR RNA (pre-crRNA) undergoes maturation and generates individual mature crRNAs. Together with a transactivating crRNA (tracrRNA), crRNA serves as a guide for the Cas protein to introduce a double-strand break on the invading DNA that is complementary to the crRNA spacer sequence. A protospacer adjacent motif (PAM) is a short nucleotide motif near the target DNA sequence and is 3 nt downstream of the cleavage site. There are 3 CRISPR/Cas systems (type I, II, and III). A PAM is necessary for the activity of Cas in type I and II systems. Cas9 from the type II system is the simplest to manipulate because it is composed of a single protein instead of a large protein complex in the other systems. The dual tracrRNA:crRNA duplex is also further simplified and engineered as a single guide RNA (sgRNA). Therefore, the CRISPR/Cas9 system requires only 2 elements to introduce a double-strand break in a target DNA: an sgRNA and the Cas9 protein. After the CRISPR/Cas9 system induces a double-strand break in genomic DNA, it can be repaired by nonhomologous end joining (NHEJ) or homology-directed repair (HDR). NHEJ is error-prone. NHEJ repairs the DNA break but also introduces small deletions or insertions at the break site. HDR is error-free and repairs the DNA break using a DNA template. This template can be either an endogenous homologous chromosomal fragment or an exogenously provided DNA template. Consequently, HDR is preferred in gene editing therapy. However, the efficiency of HDR over NHEJ is generally not high. In nondividing somatic cells such as cardiomyocytes, HDR is relatively rare. Even in mitotic cells, HDR is restricted to the S and G2 phases.^501^

In inherited diseases, genetic correction can prevent transmission of the pathogenic mutation to the next generation. Early in 2014, Eric N. Olson’s group tried to use the CRISPR/Cas9 system to correct a nonsense mutation in exon 23 of the Dmd gene in mdx mice. The mdx mouse is an established animal model for Duchenne muscular dystrophy (DMD), an inherited X-linked disease.^502^ DMD is characterized by progressive weakness in muscle, including the myocardium, due to a lack of functional dystrophin protein. Death in DMD patients is usually caused by breathing complications or cardiomyopathy.^503^ In this study, Cas9, an sgRNA targeting the mutant Dmd locus, and the HDR template were injected into mouse zygotes. The editing produced mosaic animals containing 2 to 100% correction of the Dmd gene. However, even a low rate of gene correction significantly rescued the muscle phenotype. As an early attempt at gene editing using CRISPR/Cas9, some concerns were also revealed in this study. For example, although the sgRNA was designed against the mutant Dmd allele, sequencing showed that this system also created a double-strand break in the nonmutant allele in wild-type mice. Moreover, highly varied mosaicism is not acceptable in humans. Recently, a breakthrough was made in human embryos.^504^ Mutations in the thick filament-associated cardiac myosin-binding protein C (MYBPC3) gene account for approximately 40% of all genetic defects in hypertrophic cardiomyopathy.^505^ Sperm from an adult male patient with hypertrophic cardiomyopathy caused by a heterozygous dominant 4-bp GAGT deletion in exon 16 of MYBPC3 and oocytes from healthy female donors were studied. A mixture of sgRNA, recombinant Cas9 protein and DNA template was injected with sperm into MII phase oocytes. Out of 58 analyzed embryos, 16 (27.6%) were uniformly heterozygous carrying NHEJ-induced alleles. The remaining 42 embryos were uniformly MYBPC3WT/WT, with 41 repairing their DNA break by HDR using the maternal wild-type allele. Among these embryos, some blastomeres underwent HDR using the maternal chromosome as a template, while others used the exogenously provided DNA as a template. Further whole genome sequencing did not identify any off-target mutations. The M-phase injection approach proposed by this study achieved a 100% targeting efficiency and a dramatic decrease in the rate of mosaicism, taking a great step toward clinically practical gene editing.

An even more attractive application of the gene editing technique is to cure patients with diagnosed inherited cardiomyopathies. In vivo somatic genome editing is more ethically acceptable because the modified DNA will not be passed to the next generation. However, this method is also more challenging. As most somatic cells, including cardiomyocytes, do not proliferate, HDR is uncommon, which limits the efficiency of error-free editing. Delivery of editing tools to the target cells in vivo is also difficult, but thanks to the discovery of AAV, this issue is partially resolved in certain cell types, including skeletal and cardiac muscle. To date, most in vivo gene editing studies for cardiomyopathy have focused on DMD using AAV8 or AAV9 as delivery tools. Dystrophin is a large protein, and many regions are dispensable.^503^ Skipping mutant exons or nearby exons can bypass nonsense mutations and restore the open reading frame of the Dmd gene. It is estimated that 80% of DMD patients can benefit from exon skipping.^506^ Most of the published studies aimed to restore dystrophin expression using this strategy. For DMD caused by exon 50 deletion, a CRISPR/Cas9-induced single cut at the exon 51 splice acceptor site skipped exon 51 or introduced an in-frame insertion.^507,508^ For DMD caused by a point mutation, 2 cuts at the flanking introns removed the nonsense exon with or without nearby exons.^509–513^ These studies all reported high efficiency in restoring dystrophin expression and skeletal and cardiac muscle function. Long-term evaluation further confirmed that in vivo gene editing in mdx mice was well tolerated, and the mice had sustained dystrophin restoration, although the host response and unintended genome modifications were also documented.^514^ There were also attempts to cure autosomal dominant inherited cardiomyopathy. A gain-of-function missense mutation in the RYR2 (ryanodine receptor 2) gene can promote uncontrolled calcium leakage from the sarcoplasmic reticulum and cause catecholaminergic polymorphic ventricular tachycardia (CPVT). Mutant alleles can be disrupted by one double-strand break induced by the AAV9-delivered CRISPR/Cas9 system. The treated mutant heterozygous mice were free from arrhythmias, while 71% of the untreated control developed arrhythmias. The enhanced Ca^2+^ signal was also normalized by gene editing.^515^ A similar strategy also worked for mice with PRKAG2 cardiac syndrome.^516^

Recently, the results of a phase 1 clinical trial aiming to treat hereditary transthyretin amyloidosis by in vivo gene editing were published.^517^ Hereditary transthyretin amyloidosis is a rare disease and is estimated to occur in approximately 50,000 individuals worldwide.^518^ This disease has an autosomal dominant inheritance pattern.^519^ Affected patients have amyloid polyneuropathy, cardiomyopathy, or a combination of both.^520^ A pathogenic mutation in transthyretin favors amyloidogenesis.^520^ As most circulating transthyretin is produced in the liver,^521^ the study used lipid nanoparticles with liver tropism as delivery vehicles. An mRNA encoding Cas9 protein and an sgRNA targeting the human transthyretin gene were loaded into the nanoparticles. After being infused into the circulation, plasma apolipoprotein E binds to the surface of lipid nanoparticles. The nanoparticle and its cargo are then taken up by hepatocytes through the low-density lipoprotein receptor.^522^ Six patients were treated with a low or a high dose. At Day 28 after the single-dose infusion, the lower-dose group had an average reduction in serum transthyretin of 52%, while the higher-dose group had an average reduction of 87%. Although the results were preliminary, this study provided important evidence of the therapeutic utility of CRISPR/Cas9-based in vivo genome editing in humans.

While progress on in vivo genome editing is promising, we should examine its limitations and future challenges to overcome. The in vivo studies mentioned above involved error-prone NHEJ for DNA repair. Therapeutic value is based on the condition that the downregulation of the target gene or deletion of a certain region of the target protein does not cause severe consequences. What’s more, a study published in 2019 indicated that AAV vector was commonly integrated into the DNA breaks, which could lead to potential risk.^523^ Therefore, this therapy is still restricted to highly selective gene defects. To cover a broader range of inherited cardiomyopathies, error-free editing is needed. To this end, a base editor was developed to correct pathogenic base substitutions. The Cas9 protein has been engineered to deactivate its catalytic domains while retaining its ability to bind to the target DNA sequence (dCas9). An enzyme that converts C–G to T–A^524^ or T-A to C-G^525^ is fused with dCas9 to edit the target genome base. Zeng et al. successfully corrected a Marfan syndrome pathogenic point mutation in human embryos with the base editor system.^526^ It is noteworthy that base editing does not involve DNA cleavage and repair and should enable error-free editing in both dividing and nondividing cells. This technology may have potential for in vivo correction of cardiomyopathy-associated point mutations in the future.

### Therapeutic strategies based on cell therapy

**Fig. 6:**
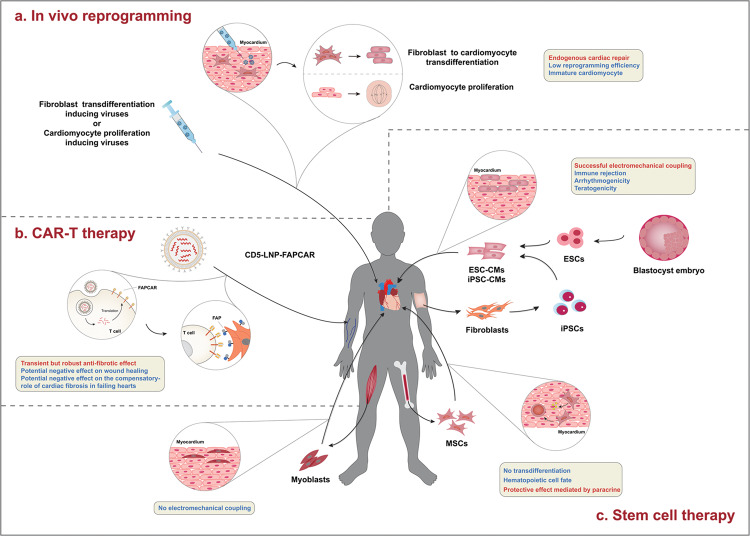
Summary of therapeutic strategies based on cell therapy for failing hearts. The earliest attempt is myoblast transplantation to the injured heart. However, these cells don’t form connection with neighboring cardiomyocytes. Transplantation of bone marrow derived mesenchymal stem cell (BMSC) shows modest benefit, which is mediated by paracrine factors secreted by BMSC instead of transdifferentiation into cardiomyocytes. Transplantation of inducible pluripotent stem cell derived cardiomyocytes (iPSC-CM) or embryonic stem cell derived cardiomyocytes (ESC-CM) successfully achieve electromechanical coupling between the transplanted and in situ cardiomyocytes, but these therapies have the shortcomings of immune rejection, arrythymogenicity, and teratogenicity. In vivo reprograming tries to induce fibroblast-to-cardiomyocyte transdifferentiation or cardiomyocyte proliferation by gene manipulation to compensate for cardiomyocyte loss. Chimeric antigen receptor (CAR) T cell therapy targeting activated fibroblast has been just proposed by a proof-of-concept study. These CAR T cells can be induced by injection of modified RNA loaded in T cell directed lipid nanoparticles. LNP: lipid nanoparticle; FAP: fibroblast activation protein; iPSC: induced pluripotent stem cell; ESC: embryonic stem cell; MSC: mesenchymal stem cell

The loss of functional cardiomyocytes is the most important reason for chronic heart failure. Replacing the injured or lost cardiomyocytes exogenously or endogenously can potentially restore cardiac function (Fig. 6).

#### Therapeutic strategies based on stem cell therapy

The earliest attempt to provide exogenously derived myocytes was made with skeletal myoblasts. In animal models of myocardial infarction, autologous transplantation of skeletal myoblasts into the infarcted area partially preserved cardiac function and attenuated ventricular remodeling.^527–529^ A phase I clinical trial with 10 patients with ischemic cardiomyopathy demonstrated that skeletal myoblast transplantation reduced the risk of hospitalization for heart failure and improved the NYHA class and LVEF.^530^ However, a multicenter, randomized, placebo-controlled, double-blind, phase II trial with 94 patients with myocardial infarction and left ventricular dysfunction showed that myoblast transplantation did not improve cardiac function, as measured by echocardiography at 6 months. The risk of arrhythmic events was increased in patients treated by myoblast transplantation.^531^ Although myoblasts have contractile features, they cannot form electromechanical coupling with nearby cardiomyocytes, which may explain the failure to improve coordinated cardiac contraction. Moreover, uncoupled myoblasts may also serve as ectopic triggers of arrhythmias. These shortcomings limit further translational potential of this strategy.^532^

In the microenvironment in the myocardium, stem cells may be able to transdifferentiate into functional cardiomyocytes that can form electric connections with surrounding cells. Bone marrow-derived mesenchymal stem cells (BMSCs) are relatively easily available. Culturing patient-derived MSCs in medium with 5-azacytidine converts them into cells with stick-like morphology and myotube-like structures.^533^ Rat BMSCs were cocultured with cardiomyocytes, and some BMSCs showed features of cardiomyocytes, including regular contraction, expression of cardiomyocyte markers, sarcomere formation and inward rectifier potassium current.^534^ These data indicated that BMSCs could also transdifferentiate into cardiomyocytes in vivo. Indeed, BMSC transplantation in a mouse model of myocardial infarction showed beneficial effects.^535,536^ These promising preclinical results led to multiple clinical trials testing BMSC transplantation in chronic heart failure and myocardial infarction. The REPAR-AMI trial showed that among patients with myocardial infarction, BMSC intracoronary infusion after reperfusion therapy improved LVEF and reduced the risk of mortality, reinfection, and revascularization procedure.^537^ In the BOOST trial, which had similar settings, intracoronary infusion of BMSCs did not provide long-term benefits on LV systolic function but promoted short-term LVEF recovery after reperfusion therapy.^538^ In the C-CURE trial, BMSCs were first exposed to a cardiogenic cocktail. Then, the derived cardiopoietic stem cells were delivered by endomyocardial injection to patients with chronic ischemic heart failure. The treatment group had significantly more improvement in terms of LVEF and 6-min walk distance than the control group.^539^ The MSC-HF trial showed that intramyocardial injection of BMSCs reduced left ventricular end-systolic volume in chronic ischemic heart failure.^540^ The POSEIDON trial demonstrated the safety and efficacy of both allogeneic and autologous BMSC transplantation.^541^ However, some other studies showed inconsistent results. The TIME and the FOCUS-CCTRN trials included more than 100 patients. The TIME trial did not show a beneficial effect of BMSC therapy on myocardial infarction in terms of the recovery of global or regional left ventricular function,^542^ while the FOCUS-CCTRN trial did not show a benefit on chronic ischemic heart failure in terms of cardiac function, exercise capacity, or cardiac perfusion.^543^ A meta-analysis summarized the results from 50 clinical trials and suggested that BMSC therapy did provide modest long-term improvements in left ventricle function and reduced mortality risk in patients with ischemic heart disease.^544^ Although the results were not universally consistent, these clinical trials suggested that BMSC therapy did provide significant yet modest benefits. Moreover, the results also demonstrated the safety and feasibility of stem cell therapy in cardiovascular disease.

The turning point in BMSC therapy came with the publication of several studies that used different BMSC tracing techniques.^545–547^ These studies indicated that BMSCs transplanted in injured or normal hearts maintained a hematopoietic cell phenotype and did not transdifferentiate into cardiomyocytes in vivo. The observed clinical benefit was possibly mediated by the paracrine functions of BMSCs that regulated cardiac remodeling or angiogenesis. Most of the beneficial factors are intercellularly transferred by extracellular vesicles.^548^ For example, VEGF, hepatocyte growth factor (HGF), and IGF-1 were secreted by BMSCs and could promote angiogenesis and reduce collagen deposition.^549,550^ Secreted hypoxia- and Akt-induced stem cell factor (HASF) could activate the IGF-1 receptor, stimulate cardiomyocyte proliferation, and inhibit apoptosis.^551–554^ sFRP2 inhibited the Wnt pathway and apoptosis and protected the heart during myocardial infarction.^555^ Some paracrine factors could protect the heart by modulating metabolism.^556,557^ There were also factors that regulated the immune environment in the injured myocardium. Bone marrow mononuclear cells produced IL-10, which reduced T cell accumulation and therefore inhibited inflammation in infarcted hearts.^558^ Paracrine factors from MSCs induced the secretion of SDF-1 and plasminogen activator inhibitor-1 (PAI-1) by macrophages. Both factors induced EC differentiation, which promoted angiogenesis.^559^ As most of the beneficial effects of BMSC therapy come from paracrine effects, purified extracellular vesicles from BMSCs might have improved efficacy and avoid unnecessary side effects.

Another cell therapy approach was the transplantation of differentiated cardiomyocytes. Embryonic stem cells (ESCs) are derived from the inner cell mass of a blastocyst. ESCs are characterized by pluripotency, self-renewal, and rapid proliferation. ESCs can be induced to differentiate into both atrial and ventricular cardiomyocytes in vitro.^560^ The transplantation of ESC-derived cardiomyocytes (ESC-CMs) improved cardiac function after myocardial infarction in rodent models.^561,562^ Similar beneficial effects were also observed in nonhuman primate models of cardiac I/R injury. Remuscularization could be observed in the infarcted region. Most importantly, transplanted cardiomyocytes form electromechanical couplings with the nearby myocardium.^563,564^ Unlike BMSC therapy, transplanted ESC-CMs contracted synchronously with the host’s myocardium and reduced ventricular arrythmias caused by myocardial injury.^565^ However, ESC-CM therapy has 2 major shortcomings: the requirement of immunosuppression therapy to inhibit rejection and the risk of teratoma due to the immaturity of ESC-CMs. The discovery of inducible pluripotent stem cells (iPSCs) provided an alternative approach for cell therapy. The overexpression of Yamanaka factors (Oct3/4, Sox2, Klf, and c-Myc) reprograms mature somatic cells into iPSCs,^566,567^ which can be induced to differentiate into various cell types, including cardiomyocytes.^568^ The transplantation of human cardiomyocytes derived from iPSCs (iPSC-CMs) remuscularized the infarct region and improved cardiac function in a nonhuman primate model of myocardial infarction.^569^ Theoretically, iPSC-CM transplantation should not induce rejection because it is autogeneic. However, this is not the case. Zhao et al. showed that autogeneic iPSCs were rejected in C57BL/6 mice.^570^ A subsequent study showed that during dedifferentiation from somatic cells to iPSCs, a mutation occurred in mitochondrial DNA (mtDNA), which could elicit an immune response in humans and mice.^571^ Therefore, screening mtDNA in iPSCs before transplantation is important for safety. Another problem is arrhythmogenicity. iPSC-CM transplantation increased the risk of ventricular arrhythmias in nonhuman primates, which was transient and nonlethal.^569^ Much improvement is still needed before iPSC-CM transplantation can be tested in clinical trials.

#### Therapeutic strategies based on in vivo cardiac reprogramming

An endogenous myocardial repair strategy may be able to overcome the immunogenic and arrhythmogenic issues associated with exogenous cardiomyocyte replacement. New cardiomyocytes can come from existing cardiomyocytes or noncardiomyocytes through reprogramming, which represents 2 different strategies.

The first strategy is the induction of the transdifferentiation of noncardiomyocytes into cardiomyocytes. Fibroblasts are the best candidate because they are the most abundant proliferative cell type in the mammalian heart.^572–574^ Overexpression of Gata4, Mef2c, and Tbx5 (GTM) with or without Hand2 induces fibroblast transdifferentiation into cardiomyocyte-like cells that express cardiomyocyte genes, have sarcomere structures, and exhibit spontaneous beating and calcium oscillations.^574,575^ In vivo transdifferentiation was also successful. GTM retrovirus with GFP successfully infected proliferative noncardiomyocytes, especially fibroblasts, after myocardial injection. The infected cells were reprogrammed to become cardiomyocyte-like cells with action potentials, responses to electrical pacing, and electrical coupling. Delivery of GTM reduced the infarcted area and improved cardiac function in the myocardial infarction model.^576,577^ Delivery of a cocktail of miRNAs (miR-1, miR-133, miR-208, and miR-499) was also able to activate cardiomyocyte genes in fibroblasts and improve systolic function after myocardial infarction.^578,579^ To avoid potential side effects of virus-based delivery, Cao et al. successfully reprogrammed fibroblasts to become cardiomyocytes with 9 small-molecule compounds.^580^ This small-molecule compound-based strategy was also effective in reducing scar formation and improving cardiac function in a myocardial infarction model.^581^ Transdifferentiation does not involve a stem cell stage and therefore should not have immunogenic or teratogenic issues. However, there are still problems that need to be overcome. The efficiency of in vivo reprogramming is rather low (5 to 15%) in fibroblasts in mice. Reprogramming is even less efficient and slower in humans. This low efficiency may lead to immature cardiomyocytes and dysfunctions in intracellular electrical and biological communication.^582^ Although benefits have been demonstrated in rodents, evidence in large animals is still needed.

The second strategy is turning the cell cycle on in cardiomyocytes. In human, there is self-renewal in the myocardium. At the age of 20, 1% of cardiomyocytes are renewed each year. The percentage decreases with age.^583^ Cardiomyocytes are proliferative in neonatal mice. However, they lose their proliferative capacity at Day 7 after birth.^584^ Cardiomyocytes from pigs also showed the same trend.^585,586^ Therefore, reprogramming adult cardiomyocytes back to their fetal state may induce their proliferative capability. Modulating cell cycle related factors is effective. Deletion of Meis homeobox 1 (MEIS 1) reactivated cardiomyocyte mitosis in adult mice without deleterious effects on heart function.^587^ The overexpression of a combination of cell cycle factors (4 F) [cyclin-dependent kinase 1 (CDK1), CDK4, cyclin B1, and cyclin D1] induces proliferation in postmitotic cardiomyocytes from mice, rats, and humans. In vivo reprogramming with the 4 F combination drove 15–20% of infected cardiomyocytes to return to the cell cycle and improved cardiac function after myocardial infarction.^588^ The Hippo pathway is a negative regulator of cell proliferation and growth. Genetic inhibition of the Hippo pathway increased cardiomyocyte proliferation and preserved heart function after myocardial infarction.^589^ Another strategy is to modulate stemness-related factors in adult cardiomyocytes. In vivo expression of Yamanaka factors in adult cardiomyocytes induced dedifferentiation and regenerative capacity. Transient expression of Yamanaka factors induced a fetal gene program in adult cardiomyocytes, while extended expression led to reprogramming and tumor formation. In a model of myocardial infarction, short-term expression of Yamanaka factors did not affect inflammation or the rate of cardiomyocyte death but increased the rate of proliferative cardiomyocytes and reduced the infarcted area.^590^

#### Therapeutic strategies based on chimeric antigen receptor (CAR) T cell therapy

Although most of the attention in cardiac cell therapy focuses on the stem cell therapy and in vivo reprogramming, it is worth mentioning that Jonathan A. Epstein’s team proposed a CAR T cell-based anti-fibrosis therapy.^591,592^ After isolated from human blood, T cells were transduced with a gene coding the CAR, an engineered receptor protein that enable T cells to target a specific protein. The main focus of CAR T cell therapy is its anti-cancer potential. T cells are engineered to recognize a protein on the surface of a tumor. After recognizing the tumor cell, CAR T cells become activated and attack these tumor cells.^593^ Epstein’s group found that fibroblast activation protein (FAP) was robustly expressed in cardiac fibroblast in diseased hearts. However, in normal hearts, FAP expression was minimal. These features made FAP an ideal target for activated fibroblasts. Mouse T cells that express a CAR construct specific to mouse FAP was established by viruses in vitro. Cardiac fibrosis was induced by 4 weeks of AngII/PE infusion. Two dosages of CAR T cell transfer at 1- and 2-week after AngII/PE initiation significantly reduced cardiac fibrosis at 4- and 8-week. Both systolic and diastolic function were improved. No toxicities were observed.^591^ This study provided the possibility for the CAR T cell therapy to prevent cardiac fibrosis. However, the persistence of the engineered CAR T cells post threat to other injuries as fibroblast activation is required for wound healing. The same research group proposed an in vivo approach for anti-FAP CAR T cell generation. Modified mRNA encoding CAR against FAP was loaded in lipid nanoparticles (LNPs) (LNP-FAPCAR). The LNPs were decorated by anti-CD5 antibodies for T cell specific uptake of the cargo (CD5/ LNP-FAPCAR). One week after AngII/PE initiation, mice received CD5/LNP-FAPCAR injection, which produced FAPCAR + T cells at 48 h. FAP trogocytosis was observed, suggesting CD5/LNP-FAPCAR successfully produced functional FAPCAR T cells. Cardiac fibrosis was reduced by CD5/LNP-FAPCAR treatment and cardiac function was improved.^592^ Unlike in vitro virally engineered FAPCAR T cells, CD5/LNP-FAPCAR generated FAPCAR T cells transiently, thus reducing the threat to fibroblast dysfunction in the future. Moreover, this in vivo method did not involve in vitro processes of T cell isolation, culture, transfection, expansion, and re-administration. These results provided a promising therapy to inhibit pathological fibrosis right after acute injury. These proof-of-concept studies proposed a promising cell therapy for cardiac fibrosis.

### Xenotransplantation

Heart transplantation remains the ultimate curative therapy for advanced chronic heart failure. However, human donor hearts are in short supply. In the United States, more than 7% of patients on the heart transplant wait list die because of the lack of availability of a suitable human donor heart.^594^ To address this large unmet need, xenotransplantation, or transplantation between different species, has been introduced. This strategy has potential as an unlimited and prompt supply of functional organs. Pigs have been chosen as the most suitable sources of xenografts because of their short gestation time, rapid growth and sexual maturity, and organ size compatibility. In contrast, although some primates are concordant without immunological barriers or hyperacute rejection, they normally have a slow natural breeding cycle and raise concerns about ethical issues.^595^ A major breakthrough was reported in pig-to-baboon life-supporting cardiac xenotransplantation in 2018.^596^ Using heart from genetically modified pigs, nonischemic continuous perfusion of an oxygenated solution of blood and nutrients, immunosuppression treatment, and anti-heart-growth treatment, Längin et al. reported a high level of transplant survival, with 4 out of 5 animals surviving for more than 3 months. In 2000, The International Xenotransplantation Association and International Society for Heart and Lung Transplantation suggested that the survival rate of animals at 3 months be at least 60% in a series of consecutive life-supporting experiments, with a minimum number of 10 nonhuman primates surviving for this period of time before considering a clinical trial.^597^ Obviously, the Längin group’s procedure represents a large step toward clinically practical cardiac xenotransplantation. We will review the obstacles that have been met and breakthroughs that have been achieved in recent decades.

#### Genetic modifications

When a wild-type pig organ is transplanted into a nonhuman primate, the graft fails rapidly within minutes to hours, which is a process called hyperacute rejection. Wild-type pigs have several carbohydrates that are absent in humans. Preformed antibodies in human blood against these epitopes drive hyperacute rejection after the xenotransplantation of hearts from wild-type pigs.^598^ Among these carbohydrates, galactose-a-1,3-galactose is targeted by 80~90% of preformed antibodies.^599^ Genetic deletion of galactose-a-1,3-galactosyl-transferase (GalTKO) can eliminate the expression of galactose-a-1,3-galactose.^600,601^ N-glycolylneuraminic acid, which is produced by cytidine monophospho-N-acetylneuraminic acid hydroxylase, and sda, which is produced by b-1,4-N-acetylgalactosaminyltransferase, were shown to be responsible for a large proportion of the residual preformed antibodies.^602–604^ Triple deletion of these 3 genes may further reduce the risk of rejection.^605–607^ Complement activation is also an important step in xenograft rejection. In humans, complement regulatory proteins (hCRPs) can downregulate complement system activity. Although similar CRPs also exist in pigs, they are not sufficient to inhibit the complement system in humans during xenotransplantation. Introduction of hCRPs, such as hCD46,^608,609^ hCD55,^610,611^ or hCD59,^612^ reduces complement-mediated graft injury. Combined with immunosuppressive therapy, GalTKO or the expression of one or more hCRPs dramatically reduced hyperacute rejection and prolonged the graft survival time in heterotropic nonlife supporting cardiac xenotransplantation.^613^ The combination of GalTKO and hCD46 expression further prolonged nonlife-supporting xenograft survival to 236 days.^614^

As the graft survival time increases, coagulation dysregulation becomes more obvious,^615^ suggesting another major barrier to successful xenotransplantation. Under physiological conditions, there is a balance between coagulation and anticoagulation. Thrombomodulin (TBM)-protein C is part of the anticoagulation system. The interaction of TBM on the surface of the endothelium and protein C in the circulation promote the latter’s activation. Activated protein C suppresses factors Va and VIIIa and prevents thrombin formation, thereby downregulating the coagulation cascade.^616^ Unfortunately, pig TBM is incompatible with human protein C, which makes their binding ineffective.^617^ Adding hTBM to the GalTKO/hCD46 genetic background extended xenograft survival by 1 year with immunosuppressive therapy.^618,619^

#### Immunosuppressive and anti-inflammatory therapy

As mentioned above, immunosuppressive therapy is important for long-term survival of cardiac xenograft. In the case of study from Längin et al.^596^, induction regimen included anti-CD20 for B cell depletion, anti-thymocyte globulin, and either an anti-CD40 monoclonal antibody or humanized PASylated Fab-CD40L to block costimulation pathway. Maintenance therapy consisted of mycophenolate mofetil, anti-CD40 monoclonal antibody or humanized PASylated Fab-CD40L, and methylprednisolone. As inflammation also contributed to graft failure, anti-inflammatory therapy was also adopted by Längin et al., which included an IL-6-receptor antagonist, TNF inhibitor, and IL-1-receptor antagonist.

#### Nonischemic continuous organ perfusion

Compared with heterotropic nonlife supporting xenotransplantation, orthotopic life supporting xenotransplantation is even more challenging. In addition to preventing rejection, preserving normal cardiac function is also critical to successful orthotopic xenotransplantation. Perioperative cardiac xenograft dysfunction (PCXD) occurs in 40 to 60% of cardiac orthotopic xenotransplantations.^620^ It is believed that ischemia–reperfusion injury is one of the reasons for PCXD. To prevent ischemia between explanation and transplantation, a perfusion system was developed.^621^ Pig hearts were preserved at 8 °C in an oxygenated albumin-containing hyperoncotic cardioplegic solution that contained nutrients, hormones and erythrocytes. Oxygen consumption in the heart was significantly reduced in this system.^622^ Längin’s group continuously perfused and oxygenated the hearts from explanation to transplantation. During implantation surgery, the hearts were intermittently perfused every 15 min. Using this continuous perfusion system, PCXD was successfully avoided.^596^

#### Inhibition of heart overgrowth

The problem of heart overgrowth was not recognized until recent technical progress enabled orthotopic xenografts to survive long enough to observe cardiac hypertrophy and diastolic dysfunction.^596^ Previous studies on kidney and lung xenografts indicated that graft overgrowth after transplantation depended on intrinsic factors.^623^ However, Längin’s group found that the situation was more complex in cardiac xenografts. The researchers successfully prevented cardiac overgrowth with a combination of 3 treatments. First, recipient baboons received antihypertensive treatments to lower the blood pressure to the donor pig’s level. Second, given that cortisone can cause cardiac hypertrophy, recipients were weaned from cortisone at an early stage. Third, pharmaceutical inhibition of the cell growth regulator mTOR was achieved with sirolimus. These results indicate that cardiac overgrowth depends largely on host factors and is amenable to medical treatment.

During the preparation of this review, a piece of exciting news comes from University of Maryland Clinical Center.^624^ A life-supporting pig-to-human orthotropic heart xenotransplantation was successfully performed on patient with terminal heart disease, who was not qualified to be on the transplant list and ineligible for an artificial heart pump. The donor pig was genetically modified by knock-out of 3 genes responsible for rapid antibody-mediated rejection, knock-in of 6 human genes responsible for immune acceptance, and knock-out of 1 gene to prevent heart overgrowth. The donor heart was preserved in a perfusion device called “XVIVO heart box”. Among with conventional anti-rejection drugs, an experimental compound was also used to suppress the immune system. At the time of the report, the recipient patient was doing well for at least 3 days after surgery. Detailed information has not been revealed yet. The follow-up of this recipient will shed light on the field of cardiac xenotansplantation.

## Conclusions and future perspectives

Therapeutic improvements always depend on 2 factors: novel theoretical discovery and technical innovation. Basic research in recent decades has dramatically changed our understanding of the pathogenesis of chronic heart failure. The canonical protein-based signaling cascade is no longer the only player in the diseased heart. With the increase in studies focusing on RNA function in recent years, signal transduction in the failing heart has been shown to involve a network of protein–protein, protein-RNA, and RNA-RNA interactions. Epitranscriptome RNA modifications further add to the complexity of this regulatory network. At the cellular level, chronic heart failure is not only a disease of cardiomyocytes. Rather, it is driven by the massive dysregulation of intercellular crosstalk and interactions among cardiomyocytes, fibroblasts, immune cells, VECs, and LECs. These novel discoveries provide numerous opportunities for identifying new therapeutic targets for chronic heart failure treatments. Traditionally, chemical activation or inhibition of protein regulators is the main treatment for chronic heart failure. With the development of novel therapeutic techniques, the landscape of chronic heart failure therapy is changing. Gene therapy provides an approach for the direct manipulation of gene expression; cell therapy attempts to replace dysfunctional cardiomyocytes endogenously or exogenously; gene editing therapy has the potential to cure inherited cardiomyopathies; and xenotransplantation techniques provide unlimited functional hearts for the ultimate therapy: cardiac transplantation. With progress in both mechanistic studies and therapeutic techniques, we may be able to see a new picture of chronic heart failure management in the near future.
